# Chemometric Characterization of Strawberries and Blueberries according to Their Phenolic Profile: Combined Effect of Cultivar and Cultivation System

**DOI:** 10.3390/molecules24234310

**Published:** 2019-11-26

**Authors:** Milica Fotirić Akšić, Dragana Dabić Zagorac, Milica Sredojević, Jasminka Milivojević, Uroš Gašić, Mekjell Meland, Maja Natić

**Affiliations:** 1 Faculty of Agriculture, University of Belgrade, 11080 Belgrade, Serbia; jasminka@agrif.bg.ac.rs; 2 Innovation Center, University of Belgrade-Faculty of Chemistry, P.O. Box 51, 11158 Belgrade, Serbia; ddabic@chem.bg.ac.rs (D.D.Z.); pantelicm@chem.bg.ac.rs (M.S.); 3 Institute for Biological Research “Siniša Stanković”—National Institute of Republic of Serbia, University of Belgrade, 11060 Belgrade, Serbia; uros.gasic@ibiss.bg.ac.rs; 4 Norwegian Institute of Bioeconomy Research-NIBIO Ullensvang, NO-5781 Lofthus, Norway; mekjell.meland@nibio.no; 5 Faculty of Chemistry, University of Belgrade, P.O. Box 51, 11158 Belgrade, Serbia; mmandic@chem.bg.ac.rs

**Keywords:** *Fragaria* x *ananassa*, *Vaccinium corymbosum*, organic production, integrated production, fruit, leaf, phenolic profiles, antohocyanin, principal component analysis

## Abstract

Chemical characterizations of leaves and fruits that were obtained from organically and integrally produced strawberries (′Favette′, ′Alba′, and ′Clery′) and blueberries (′Bluecrop′, ′Duke′, and ′Nui′) from western Serbia were undertaken in this study. Phenolic analysis was done while using ultra-high performance liquid chromatography coupled to a linear ion trap-Orbitrap hybrid mass analyzer, while total phenolic content (TPC), total anthocyanin content (TAC), and radical-scavenging activity (RSA) by spectrophotometry. In general, leaves and fruits from blueberry showed higher levels of TPC and TAC as compared to strawberry. These chemical traits were larger in organic grown fruits and larger in leaves than fruits. The most abundant phenolics in leaves and fruits of blueberry was 5-*O*-caffeoylquinic acid, followed by quercetin 3-*O*-galactoside, while catechin, quercetin, and kaempferol 3-*O*-glucosid were dominant in the leaves and fruits of strawberry. *cis*, *trans*-Abscisic acid was detected in all fruit samples, but not in leaves. Blueberries (both fruits and leaves) were separated from strawberries, but only organic blueberry fruits were distinguished from integrated fruits, according to principal component analysis. Quercetin, kaempferol, 5-*O*-caffeoylquinic acid, ferulic acid, caffeic acid, catechin, *p*-coumaric acid, and *p*-hydroxybenzoic acid were the most influential phenolic compounds for the separation. Much higher contents of TPC, RSA, TAC, quercetin 3-*O*-galactoside, and quercetin were found in fruits and TPC, RSA, catechin, *p*-hydroxybenzoicacid, *p*-coumaricacid, and ferulic acid in leaves in all three blueberry cultivars and the strawberry cultivar ′Clery′. These phenolic compounds are good sources of antioxidant compounds with potentially high beneficial effects on human health.

## 1. Introduction

Cultivated strawberry (*Fragaria x ananassa* Duch.) and highbush blueberry (*Vaccinium corymbosum* L.) are considered to be economically important freshly consumed fruits, and the production volumes are rapidly increasing worldwide during recent years [1,2,3,4]. Organic and integrated productions are two most dominant fruit productions in Europe. Integrated production follows Integrated Pest Management Directive 2009/128/ EC, while organic production is implementing European Action Plan for Organic Food and Farming [Council Regulation (EC) 834/2007]. Some principles of both production systems are similar and both products are considered to be ‘premium‘ food, but as compared to organic production, where no synthetic pesticides and fertilizers are allowed, in integrated production the use of chemicals and artificial inputs are not restricted [5].

Lately, there has been an increasing interest in organic fruit production due to environmental, economic, and social concerns. Besides avoiding chemicals, it lowers soil degradation, minimizes non-recyclable inputs and the presence of pesticide residues in food, manages animals extensively with focus on their wellbeing, and preserves natural resources and the rural landscapes, thus making it environmentally friendlier, safer, and contributing to the global food security [6,7]. Berry-fruits are the fastest growing organic fruit category, with an increased area of nearly 300% in the last decade worldwide [8]. In 2017, organic berry productions were grown on 63,543 ha, with Poland, Chile, and Spain as the leading countries [9]. Some scientific studies affirmed the nutritional value of organic fruits based on their greater concentration of particularly potent phenolic compounds that are believed to be more nutritious and beneficial to human health [10,11,12].

Besides their exquisite flavor, berry-fruits have gained significant attention by consumers due to a high content of health promoting compounds based on considerable quantities of different phytochemicals. Strawberries and blueberries are both relevant sources of phenolic compounds, including both flavonoid and non-flavonoid types, which mainly represent anthocyanins, flavonols, and flavanols, followed by phenolic acids (hydroxybenzoic/hydroxycinnamic acids), as well as hydrolysable (ellagitannins and gallotannins) and some condensed tannins [4,13,14,15]. These berry-fruit phenolics are well known for their antioxidant, anti-inflammatory, antimicrobial, antihypertensive, anti-allergy, and antidiabetic properties [11,16,17,18]. Other plant parts can also have medical application and they have been used in various forms [19]. Leaves contain many bioactive compounds, including flavonoids, ascorbic acid, tannins, and essential oils, which also act as powerful antioxidants that neutralize the harmful effects that are associated with reactive oxygen species [20]. Blueberry leaves are found to be a prospective source of phenolic compounds, such as anthocyanins and chlorogenic acid, ranging from 2.321–2.636 mg of malvidin 3-glucoside/g and from 49.34–52.66 mg of chlorogenic acid/g dry matter, respectively [21]. Leaves from the *Vaccinium* species show a liver lipid-lowering effect [22], and present neuroprotective [23], hypotensive [24], and anti-diabetic activity [25]. Potentially health-promoting phenolic compounds have also been found in strawberry leaves (including gallic acid derivatives, ellagitannins, chlorogenic acids, flavonoids, and proanthocyanidins). The leaf extract of strawberries can be used to treat diabetes nephropathy by regulating thyroid hormones, which play an important role in the metabolism of glucose and lipids [26,27].

Quantitative variations in phenolic compounds are mostly associated with genetic background, particularly in its interaction with the environmental factors, cultivation systems, and growing location [2,14,28,29,30,31,32,33]. It can also differ in certain stages of plant development and fruit ripeness [3,15,34,35], showing non-uniform concentrations in all parts of the plant [36,37]. Huge variability has been recorded among different strawberry and blueberry cultivars in terms of phenolic content and, correspondingly, the expressed total antioxidant capacity [31,38,39]. Cultivation techniques can additionally influence the phytochemical content of strawberries [4,30,40] and blueberries [3,41,42]. In that regard, growing system and agricultural practices should be adjusted to the needs of a single cultivar or group of cultivars with similar requirements to enhance their bioactive potential [43].

Few studies on nutritional fruit quality contrasting organic with conventionally/integrated grown fruits are conducted with a focus on differences in phenolic composition related to the cultural systems [7,42,44]. Therefore, the main objective of this study was to evaluate how organic and integrated farming affect quantitative variations in individual phenolic compounds and their distribution in fruits and leaves, as well as the total phenolic content and corresponding antioxidant capacity of three strawberry and three blueberry cultivars.

## 2. Results and Discussion

### 2.1. Total Phenolic Content (TPC), Radical-Scavenging Activity (RSA), and Total Anthocyanin Content (TAC) Results

Some differences were noticeable between blueberry and strawberry fruits when comparing the results that are presented in Table 1. Higher contents of total phenolics and total anthocyanins were observed in blueberry fruit samples (TPC: 2.27–6.26 g GAE/kg; TAC: 0.62–2.86 g cy-3-glu/kg) than in strawberry fruits (TPC: 1.18–2.27 g GAE/kg; TAC: 0.16–0.37 g cy-3-glu/kg). These results are in line with the database URL:http://www.phenol-explorer.eu, where the TPC of high bush blueberry and strawberry fruits ranged from 0.2 to 8.68 g GAE/kg FW (based on nine references) and from 0.72 to 4.43 g GAE/kg FW (based on seven references), respectively. Interestingly, the ranges that were obtained for radical-scavenging activities in blueberries and strawberries were both similar (18.31–33.83 mmol TE/kg and 16.32–24.05 mmol TE/kg). A bit lower ranges for all three traits (TPC, RSA, and TAC) were obtained by Panico et al. [45] for two strawberry cultivars (Tudla and Maletto) and for TAC and RSA by Crespo et al. [46] for cultivars ´Antea´, ´Asis´, and ´Matis´. This is probably due to the different cultivars and agro-climatic conditions tested. All of the organically produced strawberry and blueberry fruit samples were characterised with significantly higher RSA, TPC, and TAC values when compared with integrated fruits (with the exception of strawberry Favette´ where the opposite trend was observed). This is in accordance with Ochmian et al. [7], who worked with blueberries and Olsson et al. [11], who studied strawberries, and proved that both organically grown fruit species have a higher level of TPC and TAC when compared to integrally produced fruits. This could be due to the fact that organically maintained soils have more total carbon and micronutrients, together with a greater microbial activity, which all together affect plant metabolism and biosynthesis of compounds with antioxidant activity [47].

In blueberry fruits, the highest values for TPC, RSA, and TAC were determined in organic ´Duke´ (6.26 g GAE/kg, 33.83 mmol TE/kg, and 2.86 g cy-3-glu/kg, respectively). Generally, TPC in blueberries ranged from 2.27 g GAE/kg (integrated ´Bluecrop´) to 4.38 g GAE/kg (organic ´Bluecrop´), RSA was in the range from 18.31 mmol TE/kg (integrated ´Nui´) to 25.36 mmol TE/kg (integrated ´Duke´), while the range of TAC was between 0.62 g cy-3-glu/kg (integrated ´Bluecrop´) and 1.80 g cy-3-glu/kg (integrated ´Duke´). The lowest values for all three parameters were obtained in the integrated ´Bluecrop´ and ´Nui´.

In strawberry fruits, organically grown ´Alba´ and ´Clery´ had the highest total phenolic contents (2.27 g GAE/kg and 2.04 g GAE/kg, respectively), radical-scavenging activities (24.05 mmol TE/kg and 23.35 mmol TE/kg, respectively), and total anthocyanin contents (0.37 cy-3-glu/kg and 0.33 cy-3-glu/kg, respectively). The radical-scavenging activities of strawberry leaves with a range from 802.29 to 2237.31 mmol TE/kg were significantly higher when compared with the values for blueberry leaves (range: 336.19–679.94 mmol TE/kg) (Table 2). The TPC values ranged from 46.96 to 81.06 g GAE/kg in blueberry leaves, and the range was almost the same in strawberry leaves (38.22–82.25 g GAE/kg). All of the blueberry leaf samples from organic production had slightly higher TPC and RSA values when compared with integrated samples, while the distribution of these values was unequal between the integrated and organic strawberry leaves.

In blueberry leaves, organically produced ´Bluecrop´ had the highest TPC (81.06 g GAE/kg), followed by organic ´Nui´ (77.65 g GAE/kg). Organically grown ´Nui´ also stood out with significantly higher RSA (679.94 mmol TE/kg) when compared to the rest of the blueberry leaves. This is in line with the finding of Cezarotto et al. [48], who determined that TPC in ´Alice Blue´, ´Flórida M´, ´Bluegem´, ´Clímax´, and ´Powder blue´ cultivars originated from *Vaccinium ashei*. Additionally, Wu et al. [49] determined the total phenolic content of 73 different blueberry leaves that included ´Bluecrop´ (91.80 g GAE/kg), ´Nui´ (114.6 g GAE/kg), and ´Duke´ (78.71 g GAE/kg) cultivars that were in accordance with these results for organically grown samples, or higher. The results that were obtained herein for blueberries were lower when compared to six blueberry hybrids (derived from *V. corymbosum*, *V. virgatum*, and *V. darrowii* crosses) that were examined by Grace et al. [50]. In investigated hybrids, the results were in the range: 7.53–24.5 mg GAE/g.

The organically grown strawberry cultivar ´Clery´ was characterized with the highest TPC (82.25 g GAE/kg) in the leaves. TPC was almost uniform with the range from 38.22 g GAE/kg (organic ´Alba´) to 49.89 g GAE/kg (in integrated ´Alba´) in the rest of the analysed strawberry leaves, which coincides with the results of Buřičová et al. [51]. ´Favette´ leaf samples from both growing regimes gave the highest RSA among all samples, with value of 2237.31 mmol TE/kg in organic and 1880.32 mmol TE/kg in the integrated regime.

### 2.2. Determination of Phenolic Profile using UHPLC-LTQ Orbitrap MS^4^ Technique

The investigated strawberry and blueberry extracts contained a wide range of phenolic compounds and the most abundant compounds were flavonol glycosides [16,52] and hydroxycinnamic and hydroxybenzoic acids in free, ester, and glycoside forms, as was expected [53,54]. In addition, a number of flavan-3-ols and procyanidins, as well as ellagic acid derivatives, were found in the extracts.

One of the goals of this study was obtaining insight into the metabolic profile of organic and integral strawberry and blueberry extracts while using the non-targeted approach, which resulted in the identification of total of 93 compounds (Table 3). The identification of compounds was based on the search for the [M − H]^−^ deprotonated molecule and its MS^4^ fragmentation. Table S1 shows the presence of identified compounds in organic and integral strawberry and blueberry fruit extracts, while, for the leaf extract, the presence is given in Table S2.

Hydroxycinnamic acid esters mainly quinic acid and various glycosides with characteristic fragmentation from the loss of sugar units (132 Da and 162 Da for pentosyl and hexosyl derivatives, respectively) were the most abundant compounds from the group of phenolic acids.

As was already mentioned, a large number of flavonols (kaempferol, quercetin, isorhamnetin, myricetin, and syringetin derivatives) were found in the tested fruit and leaf extracts. The determination of the glycosylation site in the case of flavonol 3-*O* or 7-*O* glycoside derivatives was achieved according to previously reported mass spectrometry rules [55,56]. Several flavonol glycosides have been identified as acyl derivatives with acetyl, malonyl, methyl-manonyl, and *p*-coumaroyl group linked to sugar moiety. Additionally, a number of hexuronyl derivatives have been identified with specific fragmentation (loss of 176 Da). For example, compound **24** at 639 *m*/*z* and 5.45 min. (Table 3) only identified in strawberry leaf samples was marked as quercetin 3-*O*-hexoside-7-*O*-hexuronide. It showed MS^2^ base peak at 463 *m*/*z* formed by specific loss of hexuronyl group (176 Da) from the 7-O position. Further, MS^3^ base peak found at 301 *m*/*z* (loss of hexosyl group-162 Da) and MS^4^ fragmentation confirmed the presence of quercetin as aglycone. At this point, it is interesting to mention compound **37** eluted at 6.14 min., which is structural isomer of compound **24** with same exact mass. It gave MS^2^ base peak at 301 *m*/*z* (mass of deprotonated quercetin), generated by the elimination of hexosyl-hexuronide moiety and secondary MS ^2^ peak at 337 *m/z*, which corresponds to a mass of disaccharide residue without water. This compound, marked as quercetin 3-*O*-hexosyl-hexuronide, was only found in strawberry leaf samples. Figure S1 depicts the proposed structure and fragmentation pathway of compound **37**.

### 2.3. Differences in Strawberry and Blueberry Phenolic Profilesfrom Fruits and Leaves

A total of 20 phenols were quantified in blueberry and 11 in strawberry fruit samples, according to the results that are presented in Table 1. Aesculin, 5-*O*-caffeoylquinic acid, epigallocatechin, epicatechin, syringic acid, apigenin 8-*C*-glucoside, quercetin 3-*O*-rutinoside, sinapic acid, and naringenin were found only in blueberries, and they could be potentially used as a marker for blueberry products. As for phenols that were identified in all fruit samples, the contents of the majority of them were higher in blueberries than in strawberries, with the exception of catechin, *p*-coumaric acid, kaempferol 3-*O*-glucoside, and cinnamic acid. 5-*O*-caffeoylquinic acid was the most abundant phenolic compound in blueberries, followed by quercetin 3-*O*-galactoside, with the average contents of 74.08 mg/kg and 24.25 mg/kg, respectively. Caffeoylquinic acids and quercetin 3-*O*-galactosideare were both synthesized in plants as a response to oxidative stresses, and they are beneficial for human health limiting atherosclerosis and carcinogenesis and anti-inflammatory activities [57,58]. Contrary to our study, Zimmer et al. [59], reported that the most abundant phenolic compound in blueberry cultivars ´Briteblue´, ´Bluegem´ and ´Woodard´ was chlorogenic acid and (was not detected in this study). Another study that was conducted in China [60] proved that ferulic acid was the most dominant compound among the phenols in blueberries. The reason for such diverse results is, most probably, genetic predisposition, and/or agroecological conditions where the study was undertaken. However, Häkkinen and Törrönen [42] and Howard et al. [61] concluded that genetics play a more significant role in the synthesis of the phenolic compounds than growing conditions. Routray and Orsat [21] suggested that abiotic factors and changes in environmental conditions during the season could explain results variations. In the case of strawberries, quantification revealed catechin, quercetin, and kaempferol 3-*O*-glucoside as the dominant compounds. Additionally, Wang and Millner [62] showed that quercetin, and kaempferol, are some of the main phenolic compounds found in strawberry fruit. Generally, both components are dominant flavonoids in fruits and effective antioxidants, playing a protective role against cardiovascular diseases and anti-inflammatory and anti-carcinogenic activities [63].

The characterization of phenols in leaves (Table 2) revealed a similar amount of phenolic compounds in blueberry (18 in total) and strawberries (17 in total). 5-*O*-caffeoylquinic acid, quercetin 3-*O*-galactoside, caffeic acid, and quercetin were the most abundant, while gallocatechin, apigenin 8-*C*-glucoside, and quercetin 3-*O*-rutinoside were exclusively found in the blueberry leaf samples. Skupień et al. [64] stated that the main substances present in blueberry (*V. corymbosum* L.) leaf extracts were caffeic acid, querectin, kaempferol, and gallic acid, while Harris et al. [65], for *V. angustifolium*, showed chlorogenic acid, epicatechin, catechin, caffeic acid, and quercetin as the main constituents of leaf extracts. According to Oszmianskiet al. [66], the most abundant phenols in leaves of native blueberry in Europe were chlorogenic acid and quercetin 3-O-glucuronide. Li et al. [67] found that caffeoylquinic acid and quercetin were the most predominant phenol groups in the blueberry leaves in China. In addition, chlorogenic acid and rutin were the major phenolic compounds in the leaves of blueberry that were cultivated in Japan. This could be clarified by statements that were given by Kaur et al. [68] and Cheel et al. [69], where the composition of secondary metabolites in a plant depend on various factors, e.g., genetics, climatic conditions, growing site, harvesting time, and others.

Besides the high level of 5-*O*-caffeoylquinic acid, quercetin and catechin, strawberry leaves were unique by the presence of some quantity of syringic acid and pinocembrin. These data are in accordance with the results of Buřičová et al. [51], where catechin was detected as the dominant compound that was present in the extracts of strawberry leaves. The contents of seven phenols (5-*O*-caffeoylquinic acid, caffeic acid, quercetin 3-*O*-galactoside, aesculin, *p*-hydroxybenzoic acid, kaempferol 3-*O*-glucoside, and quercetin) were significantly higher in all of the investigated blueberry leaves than in strawberry samples, while the opposite trend was observed in the case of *p*-coumaric acid.

### 2.4. Phenolic Profiles of Plants from Organic and Integrated Production

#### 2.4.1. Blueberry Fruits

Certain differences were found when the organic and integrated grown regimes were compared (Table 1). Blueberries from organic production stored higher levels of quercetin, apigenin 8-*C*-glucoside, and six phenolic acids, namely, 5-*O*-caffeoylquinic, *p*-hydroxybenzoic, caffeic, *p*-coumaric, sinapic, and ferulic acid. Aesculin and kaempferol were only detected in organically grown blueberries, with the average contents of 0.07 mg/kg and 0.67 mg/kg, respectively. On the contrary, the integrated fruit samples had higher amounts of two flavan-3-ols (catechin and epigallocatechin).

Cinnamic acid was detected in almost equal amounts in all blueberries, regardless of the production regimes (range from 0.03 mg/kg to 0.07 mg/kg), and the same trend was in naringenin (0.24–0.28 mg/kg). ´Nui´ blueberry from organic production was distinguished from the integrated one by higher contents of the most of the phenols. The exceptions were catechin and epigallocatechin, whose contents were higher in integrated ´Nui´.

#### 2.4.2. Strawberry Fruits

All three organically grown strawberry cultivars stored higher amounts of ferulic acid, quercetin, and quercetin 3-*O*-galactoside (Table 1). On the other hand, there was no phenol whose content was higher in all the integrated strawberry fruits. ′Alba′ was the only cultivar that was characterized with higher contents of almost all quantified phenolic compounds in organically produced fruit. Moreover, the extract of ′Alba′ stood out according to the highest contents of several phenolic acids (caffeic acid, *p*-coumaric acid, vanillic acid, ferulic acid, and cinnamic acid), and two flavonols (kaempferol and quercetin). Higher contents of some phenolic acids, such as *p*-hydroxybenzoic acid, caffeic acid, *p*-coumaric acid, and cinnamic acid, were found in ´Favette´ from integrated regime in comparison to organic.

#### 2.4.3. Blueberry Leaves

The most abundant phenolic compound was 5-*O*-caffeoylquinic acid, with the range from 1359.64 to 2380.63 mg/kg, followed by quercetin 3-*O*-galactoside and quercetin in all blueberry leaves (Table 2) (ranges: 90.72–130.20 mg/kg and 83.57–214.72 mg/kg, respectively). The levels of the two major phenols, 5-*O*-caffeoylquinic acid and quercetin 3-*O*-galactoside, were higher in all of the blueberry leaves from organic production, in comparison with the integrated regime. The same tendency was observed with three phenolic acids (*p*-hydroxybenzoic, *p*-coumaric, and vanillic acid), apigenin 8-*C*-glucoside, and kaempferol 3-*O*-glucoside. On the other hand, quercetin, which was another major phenol, was found in higher amounts in ´Bluecrop´ and ´Duke´ leaves from integrated production, and organically grown ´Nui´. By further comparison, it was observed that gallocatechin was the only phenol that was quantified in higher contents in all leaf samples from integrated production.

Organically grown ´Nui´ stored higher amounts of all phenols, with the exception of gallocatechin and catechin, when compared to the ´Nui´ leaves from integrated production. Moreover, it should be pointed out that organic ´Nui´ stood out with the highest amounts of the majority of phenols in comparison to all the other blueberry leaf samples. Organically grown ´Duke´ was also characterised with higher contents of the most of the phenolic compound (twelve) when compared to the integrated ´Duke´ samples. The opposite trend was observed with ´Bluecrop´ samples, as ten of eighteen phenolics were present in higher amounts in the integrated ´Bluecrop´ leaves.

#### 2.4.4. Strawberry Leaves

The amounts of catechin and cinnamic acid were higher in the organic leaves when compared to integrated (Table 2). On the contrary, all of the integrated samples stored higher amounts of 5-*O*-caffeoylquinic acid and aesculin in comparison to the organically grown ones, while the content of pinocembrin was fairly uniform in the samples from both regimes (range from 0.29 to 0.33 mg/kg). 5-*O*-caffeoylquinic acid stood out as the most abundant phenolic compound in strawberry leaves from integrated production, with the average concentration amounting to 168.67 mg/kg. This phenolic acid was also the dominant compound in organic ´Alba´ (92.22 mg/kg). In organically grown ´Clery´, the prevalent was catechin (31.86 mg/kg), while, in organic ´Favette´, these were catechin (30.36 mg/kg) and quercetin (32.35 mg/kg). Interestingly, the highest and lowest contents of sinapic acid were observed in ´Favette´ samples, concentration in integrated leaves amounted 27.47 mg/kg, while it was 0.04 mg/kg in organic.

### 2.5. Cis, trans-Abscisic Acid

*cis, trans*-Abscisic acid (ABA) is a hormone that is ubiquitous in plants, but it can be also found from sponges to humans [70]. In plant metabolism, it regulates plant growth and development, cell wall metabolism, responses to biotic and abiotic stresses (pests, cold, drought, salinity, UV radiation), change in gene expression, and adaptive physiological responses, bud and seed dormancy, seed and pollen germination, fruit ripening and senescence, root geotropism, stomatal functions, quality formation, sugar and acid metabolism, and phenolic metabolism [71,72,73]. ABA is a ripening promoter that influences fruit softening, aroma, anthocyanin biosynthesis, and growth in both climacteric (blueberry) and non-climacteric fruits (strawberry) [74].

In our study, ABA was detected in all fruit samples (Table 1), while its presence did not characterize the leaves. Higher amounts were observed in blueberries (climacteric fruit, which continues to ripe after harvesting) when compared with strawberries (non-climacteric fruit) and the average values were 0.84 and 0.15 mg/kg, respectively. These results were expected, as it was documented that the content of ABA in climacteric fruits increases from maturation to harvest, while it increases before maturation and decreases until harvest in non-climacteric fruits [75]. When results that were obtained for blueberries from the two growing regimes were compared, significantly higher contents of ABA were found in integrated fruits. The highest contents were detected in the ´Bluecrop´ samples, both integrated (1.40 mg/kg) and organic (1.04 mg/kg), and the lowest in cultivar ´Duke´ (integrated: 0.70 mg/kg; organic: 0.25 mg/kg). Regarding the fact that ABA is a stress hormone [76] that is synthesized in response to many kinds of stresses, it can be hypothesized that the synthesis of this hormone is cultivar dependent, concluding that ′Bluecrop′ could be a more stress sensitive cultivar. In strawberry fruits, organically grown ´Alba´ and ´Favette´ were richer in ABA when compared with integrated production.

According to Harris [77], ABA is controlling different aspects of root architecture, such as cell division and cell elongation. The root system of strawberry is very complex, having primary roots (that are mainly used for storage), secondary roots (used for water and nutrient movement), and thousands of root-hairs, which are important for water and nutrients capturing from the soil. Blueberries have a shallow and fibrous root system without any root-hairs. The synthesis of ABA is influenced by different root architecture and physiology of root growth in those two fruit species.

### 2.6. Leaf (Source)-Fruit (Sink) Relationship

The chemical analysis of leaves and fruits from organic and integrated production showed that fruits from both fruit species had much lower TPC and RSA when compared to leaves. ´Blueberry´ leaves had, on average, 19 fold higher TPC when compared to fruits, while, in strawberry, it was 30 fold higher. Similar results were obtained for RSA, where in blueberry RSA was, on average, 19 folds higher in leaves and, in strawberry, 77 folds higher in leaves when compared to fruits. Ehlenfeldt and Prior proved similar results for blueberries [78], where leaves of *Vaccinium corymbosum* cultivars had much higher phenolics values then fruits. All of the individual phenolic compounds that were present in both organs were much higher in leaves in contrast to fruits. The biggest differences were determined for caffeic acid, (up to 100 folds higher in leaves of integrated ′Nui′ as compared to fruits), quercetin 3-*O*-rutinoside (up to 46 folds higher in leaves of integrated ′Clery′ compared to fruits), 5-*O*-caffeoylquinic acid (up to 44 folds higher in leaves of organic ′Duke′ compared to fruits), and quercetin (up to 44 folds higher in leaves of integrated ′Bluecrop′ when compared to fruits). This could be explained with the fact that source-sink imbalance, which can happen with a small sink (demand) combined with a large source capacity (supply), or any other alteration in translocation between source and sink modules, can lead to the accumulation of end-products in leaf photosynthetic tissues [79,80]. The accumulation of phenolic compounds in leaves can be clarified with its role as a chemical defense against herbivore insects or some fungi, especially by knowing that quercetin and caffeic acid and its derivates are some of the most important antioxidant stress protectants for plants [81,82]. When compared fruits and leaves phenolic profiles, it should be pointed out that epigallocatechin and epicatechin were only found in blueberry fruit samples but not in leaves, while gallocatechin only in blueberry leaves. A flavanone pinocembrin that was just found in strawberry leaves has the potential to treat neurodegenerative diseases, cerebral ischemia, and atherosclerosis [83].

### 2.7. Principal Component Analysis

Principal component analysis (PCA) was used to establish similarity/dissimilarity among the phenolic profiles of blueberry and strawberry fruit and leaf samples that were grown in organic and integrated production systems.

**Figure 1:**
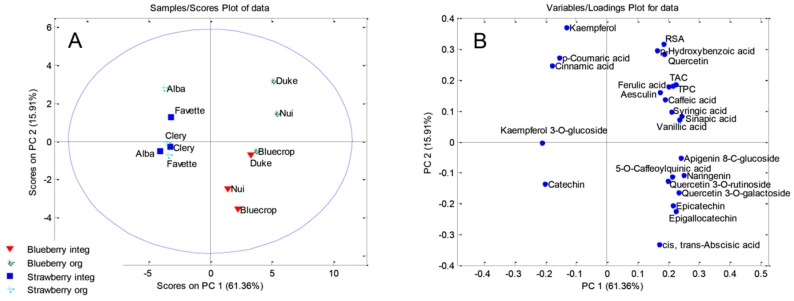
Principal Component Analysis: (**A**) score plot of the blueberry and strawberry fruit samples; and, (**B**) loadings plot of the blueberry and strawberry fruit samples.

As for fruit samples, PCA was applied on the data matrix 12 (the number of blueberry and strawberry fruit samples) × 24 (quantified phenols, *cis*, *trans*-abscisic acid TPC, TAC, and RSA) while using the covariance matrix with autoscaling. The obtained five-component model explained 95.64% of the total variance. The first two principal components accounted for 61.36 and 15.91% of total variability, respectively. The PCA score plot (Figure 1A) showed the clustering of investigated fruit samples into two groups along the PC1 axis. As expected, the strawberry fruits were separated from the blueberry fruit samples based on the significant differences in phenolic profiles. From the PCA loading plot, it was possible to identify the most influential variables that were responsible for the clustering (Figure 1B). The presence of syringic acid, sinapic acid, apigenin 8-*C*-glucoside, 5-*O*-caffeoylquinic acid, naringenin, quercetin 3-*O*-rutinoside, epicatechin, and epigallocatechin only in blueberry fruits, together with higher amounts of ferulic acid, caffeic acid, vanillic acid, quercetin 3-O-galactoside, TAC, and TPC, were the most important factors for the discrimination of blueberry samples from the strawberry fruits. On the other hand, the strawberry fruits were grouped based on their higher contents of catechin, kaempferol 3-*O*-glucoside, cinnamic acid, and *p*-coumaric acid. Interestingly, along with the expected separation of blueberries from strawberries based on a phenol profiles, PCA score plot also discriminated the organically and integrated produced blueberry fruits, and the separation was achieved along PC2. Three organically produced blueberry cultivars were characterized by higher amounts of *p*-hydroxybenzoic acid, quercetin, kaempferol, *p*-coumaric acid, aesculin, ferulic acid, and notably higher values of TPC, TAC, and RSA when compared to same blueberry cultivars that were grown in an integrated production system. Additionally, organically produced blueberry cultivars ′Duke′ and ′Nui′ stood out from the other blueberry fruits that were based on the highest contents of *p*-hydroxybenzoic acid and quercetin and the highest RSA values. Although organically and integrated strawberry cultivars were not distinguished, the sample ′Alba′ grown in organic production system was separated from the other strawberry cultivars due to its higher contents of kaempferol, cinnamic acid, and *p*-coumaric acid.

**Figure 2:**
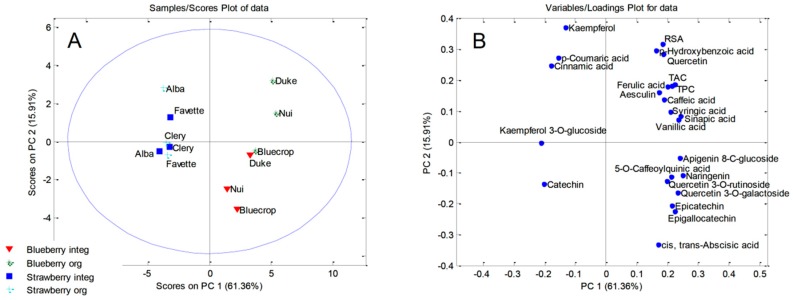
Principal Component Analysis: (**A**) score plot of the blueberry and strawberry leaf samples; and, (**B**) loadings plot of the blueberry and strawberry leaf samples.

PCA that was applied on TPC, RSA, and twenty quantified phenolics obtained for blueberry and strawberry leaf samples resulted in five-component model that explained 92.63% of total variance (PC1, PC2, PC3, PC4, and PC5 accounted for 63.49%, 13.20%, 5.81%, 5.35%, and 4.79%, respectively). Based on the PCA correlation plot, there was good discrimination between blueberry and strawberry leaf samples according to PC1 (Figure 2B). Notably higher contents of caffeic acid, kaempferol, 5-*O*-caffeoylquinic acid, aesculin, kaempferol 3-*O*-glucoside, *p*-hydroxybenzoic acid, quercetin, and quercetin 3-*O*-galactoside, as well as the presence of quercetin 3-*O*-rutinoside, apigenin 8-*C*-glucoside, and gallocatechin that were exclusively found in blueberry leaf extracts were the most important factors for separation blueberry from strawberry leaf samples. The most influential variables responsible for clustering of strawberry leaf extracts were several phenolic acids (sinapic acid, syringic acid, *p*-coumaric acid, and cinnamic acid), catechin, pinocembrin, and RSA (Figure 2B). The PCA score plot (Figure 2A) revealed that leaf samples were not clearly distinguished according to the way of cultivation (organic or integrated). However, one organically produced ′Nui′ leaf sample was separated from the other blueberry leaf extracts that were based on its higher contents of the large number of quantified phenols (Table 2).

## 3. Materials and Methods

### 3.1. Plant Material

Both integrated and organic production of three strawberry (‘Favette‘, ‘Alba‘, and ‘Clery‘) and three blueberry (‘Bluecrop‘, ‘Duke‘, and ‘Nui‘) production was organized at the Pambukovica village, municipality Ub, West Serbia. The distance between orchards is 250–300 m, so they represent the same micro-climate zone. Sandy mineral soil is used for establishing strawberry and blueberry orchards. The region has typical continental temperate climatic conditions without any extremes in temperature and rainfall. Integrated production was done according to the Integrated Pest Management Directive 2009/128/ EC, while organic production followed Serbian legislation [′Law on Organic Production′ (Official Gazette No. 30/10) and ′The Rulebook on the control and certification of organic production and organic production methods′ (Official Gazette No. 48/11)], which fully implemented the EU standards.

Strawberry orchards were planted in July 2013 in double rows on beds that were covered with black polyethylene foil. Plant spacing was 30 × 30 cm. Blueberry orchards were established with the two-year-old nursery trees that were planted in the spring of 2012 with at a spacing of 3 m × 1 m (3330 bushes ha−1). Fruits picking was done at full maturity in the middle of the harvest in the first year after planting (2014). Water quantity for strawberry irrigation during the most sensitive phases was 40 m^3^ of water/ha/per application. Blueberry irrigation was done with two laterals with 18 m^3^ (2 × 9 m^3^ of water/ha/per application) during the same growth development. Both fertilization and plant protection in organic and integrated production were done according to the standards that were already explained in our previously published manuscript [1]. In organic strawberry production, powdery mildew and gray mold rot occurred more than in integrated, while, in organic blueberry field, *Botrytis* blight and fruit rot was more frequent than in organic. The yield losses were around 40% in both organic strawberry and blueberry production compared to integrate. 

The trial was set up in a completely randomized design with three replications and five bushes/plants per replication for each cultivar and in each production system. At harvest, a sample of 20 randomly selected fruits and leaves (from each cultivar/cultivation systems/replication) from all around the bush/plant were taken and used to analyze the sugar profile. Immediately after harvesting, fruits and leaves were placed in hand-fridge, and then carried to the laboratory where they were stored in a freezer at −20 °C until chemical analysis.

### 3.2. Extraction of Phenols from Fruits and Leaves

Blueberry and strawberry fruit extracts were prepared according to modified method that was described by Natić et al. [84]. The frozen fruit samples were homogenized and 5g of each sample was extracted with 50 mL of MeOH acidified with 0.1% HCl. The extractions were carried during 1 h (on a magnetic agitator, at room temperature, in dark), in three replications. After each extraction, the extracts were filtered and the clear supernatants were collected. All of the supernatants were evaporated to dryness (reduced pressure at 40 °C) while using rotary evaporator IKA RV 8, Staufen, Germany and in residues MeOH/H_2_O (60/40, *v*/*v*) solution was added to ca. 50 mL. The extracts were then filtered through 0.45 μm membrane filters (Syringe Filter, PTFE, Supelco, Bellefonte, PA, USA).

All leaf samples were washed with water, dried for twenty days (on air, in the dark, at room temperature), and thereafter pooled and ground into a powder. The extraction of phenolics from the leaves was similar to the one described above for fruit, with some modification: about 2 g of dry leaf samples were extracted with 50 mL MeOH/H_2_O (70:30, *v*/*v*) containing 0.1% HCl [85].

### 3.3. Spectrophotometric Determinations

Total phenolic content, total anthocyanin content, and radical-scavenging activity were determined while using Folin-Ciocalteu, pH-differential, and DPPH˙methods, respectively. Pantelić et al. described the methods [86]. All of the measurements were done in triplicate.

### 3.4. UHPLC—LTQ Orbitrap MS^4^

A stock solution of a mixture of phenolics and *cis*, *trans*-abscisic acid at a concentration of 1000 ppm was prepared by dissolving standard compounds in methanol. A series of working solutions of concentrations were prepared by diluting the starting solution with the mobile phase: 0.025; 0.050; 0,100; 0.250; 0.500; 0.750; and, 1.000 ppm. The starting and working solutions were stored in the dark at 4 °C. The calibration curves were obtained by correlating the peak area with the concentration of standard solutions.

The separation of compounds was performed while using ultra-high performance liquid chromatography (UHPLC) system that consisted of an Accela 600 pump and an Accela auto-sampler (Thermo Fisher Scientific, Waltham, MA, USA). The column used for analytical separation was a Syncronis C18 column (50 × 2.1 mm, 1.7 μm particle size, Thermo Fisher Scientific). The mobile phase consists of (A) water with 0.1% formic acid and (B) acetonitrile with 0.1% formic acid. The gradient program was as follows: 0.0–1.0 min., 5% B; 1.0–14.0 min., 5–95% B; 14.0–14.1 min., 95–5% B; for the next 6 min., 5% B. The injection volume for all of the samples was 5 µl and the flow rate was 275 µL/min.

The liquid chromatography system was coupled to a linear ion trap-Orbitrap hybrid mass analyzer (LTQ OrbiTrap XL, Waltham, MA, USA). The ionization of compounds was performed in negative mode while using an electron-spray interface (HESI-II, Thermo Fisher Scientific). The ion source parameters were as previously described in Vasić et al. [87]. The mass spectra was recorded in the range of 100 to 1000 *m/z*. Collision-induced dissociation (CID) was used to study the fragmentation of the tested compounds. The normalized collision energy of the CID was constant (35 eV).

The compounds were quantified according to the exact mass search method (±5 ppm) based on comparison of retention time and high-resolution accurate mass (HRAM) with that of available reference standards. The results were expressed as mg/kg. The software ThermoXcalibur 2.2 (Thermo Fisher Scientific, Waltham, MA, USA) and it was employed to process the UHPLC-MS data. The exact masses of the identified compounds that were obtained by high resolution mass spectrometry (HRMS, Waltham, MA, USA) was compared with the exact masses calculated while using Chem Draw software, Waltham, MA, USA. Thus, molecular formulas of unknown compounds were obtained, while their identification was suggested based on specific MS^4^ fragmentation.

### 3.5. Statistic Analysis

All the data were expressed as the mean values of triplicate measurements. Tukey′s test was performed to detect the significance of differences (*p* ≤ 0.05) while using the statistical program NCSS (www.ncss.com). Principal Component Analysis was carried out using the PLS_Tool Box software package for MATLAB (Version 7.12.0), Budapest, Hungary. All of the data were group-scaled prior to PCA. The singular value decomposition algorithm (SVD) and a 0.95 confidence level for Q and Hotelling T2 limits for outliers were chosen.

## 4. Conclusions

High amounts of TPC, TAC, and RSA were detected from the blueberry and strawberry fruit and leaf extracts, which were grown in both an integrated and organic way. The most abundant phenolic compounds in blueberries were 5-*O*-caffeoylquinic acid, followed by quercetin 3-*O*-galactoside, while catechin, quercetin, and kaempferol 3-*O*-glucosid were the most dominant in strawberries. This suggests an importance of the genotype in determining the fruit and leaves composition of bioactive compounds.

The levels of TPC and RSA, and some individual phenolic compounds (caffeic acid, quercetin 3-*O*-rutinoside, 5-*O*-caffeoylquinic acid, and quercetin) in leaves as compared to fruits (of both fruit species) were much higher. This indicates that blueberry and strawberry leaves are an excellent source of antioxidants. Having in mind that large amounts of blueberry and strawberry leaves are discarded every year and while considering the high cost associated with growing of this species, the use of their leaves can be advantageous for the producers. Collected leaves could be strongly beneficial, as a high added-value bioactive material for various antioxidant applications in food processing, pharmaceutical, and nutraceutical industry.

Almost all organic fruits and leaves of both fruit species had higher level of TPC; TAC, RSA, and many other phenolic compounds when compared to integrally produced fruits and leaves and it promotes a more nutritious product. It is also very hard to recommend one cultivar, because a genotype with exceptional biochemical content in one production system might poorly perform in another under different agro-ecologic conditions. In temperate climatic conditions of south-east Europe, or similar, fruits of all three studied blueberry cultivars (′Bluecrop′, ′Duke′, and ′Nui′) and strawberry cultivar ′Clery′ from organic production, together with leaves of strawberry cultivars ′Alba′ and ′Favette′ from integrated production, can be a good source of bioactive compounds important from a health perspective.

## Figures and Tables

**Table 1:** Contents of quantified phenolics (mg/kg) *, *cis*, *trans*-abscisic acid (mg/kg) *, Total Phenolic Content (TPC) (g GAE/kg) *, Radical-Scavenging Activity (RSA) (mmol TE/kg) *, and Total Anthocyanin Content (TAC) (g cy-3-glu/kg) * in fruits from three blueberry cultivars and three strawberry cultivars, West Serbia in 2014.

Compound Name	Blueberry Fruits						Strawberry Fruits					
	Integrated			Organic			Integrated			Organic		
	Bluecrop	Duke	Nui	Bluecrop	Duke	Nui	Alba	Favette	Clery	Alba	Favette	Clery
**Aesculin**	0.00	0.00	0.00	0.06 ^b,^**	0.03 ^c^	0.11 ^a^	0.00	0.00	0.00	0.00	0.00	0.00
**5-*O*-Caffeoylquinic Acid**	89.04 ^c^	33.08 ^f^	48.05 ^d^	120.75 ^a^	33.73 ^e^	119.84 ^b^	0.00	0.00	0.00	0.00	0.00	0.00
**Epigallocatechin**	3.99 ^a^	2.53 ^d^	3.00 ^b^	2.92 ^c^	2.10 ^f^	2.31 ^e^	0.00	0.00	0.00	0.00	0.00	0.00
**Catechin**	2.32 ^e^	1.27^i^	1.59 ^g^	1.43 ^h^	0.67 ^j^	1.45 ^h^	3.19 ^b^	1.70 ^f^	2.52 ^c^	2.33 ^e^	2.43 ^d^	3.79 ^a^
***p*-Hydroxybenzoic Acid**	0.54 ^f^	0.80 ^c^	0.64 ^e^	0.74 ^d^	0.99 ^a^	1.00 ^a^	0.32 ^i^	0.96 ^b^	0.51 ^g^	0.73 ^d^	0.45 ^h^	0.45 ^h^
**Caffeic Acid**	0.95 ^c^	0.61 ^d^	0.42 ^f^	2.39 ^a^	1.26 ^b^	2.36 ^a^	0.33 ^h^	0.45 ^e^	0.38 ^g^	0.96 ^c^	0.22 ^i^	0.34 ^h^
**Epicatechin**	0.35 ^a^	0.30 ^b^	0.15 ^e^	0.17 ^d^	0.14 ^f^	0.22 ^c^	0.00	0.00	0.00	0.00	0.00	0.00
**Syringic Acid**	0.34 ^d^	0.90 ^b^	0.42 ^c^	0.25 ^e^	1.27 ^a^	0.43 ^c^	0.00	0.00	0.00	0.00	0.00	0.00
**Apigenin 8-*C*-Glucoside**	0.73 ^d^	0.57 ^f^	0.75 ^c^	1.28 ^b^	0.70 ^e^	1.38 ^a^	0.00	0.00	0.00	0.00	0.00	0.00
**Quercetin 3-*O*-Rutinoside**	2.29 ^d^	0.95 ^e^	3.31 ^b^	2.53 ^c^	0.81 ^f^	4.65 ^a^	0.00	0.00	0.00	0.00	0.00	0.00
***p*-Coumaric Acid**	0.14 ^j^	0.13 ^k^	0.08 ^l^	0.16 ^i^	0.26 ^h^	0.56 ^g^	0.77 ^e^	3.64 ^b^	1.05 ^d^	5.27 ^a^	0.59 ^f^	2.12 ^c^
**Quercetin 3-*O*-Galactoside**	30.92 ^a^	25.39 ^c^	18.67 ^f^	30.07 ^b^	21.15 ^d^	19.33 ^e^	0.98 ^i^	0.38 ^k^	0.21 ^l^	1.19 ^h^	0.46 ^j^	1.26 ^g^
**Vanillic Acid**	0.13 ^f^	0.27 ^a^	0.11 ^e^	0.15 ^d^	0.23 ^b^	0.21 ^c^	0.05 ^h^	0.06 ^g^	0.04 ^i^	0.07 ^g^	0.05 ^h^	0.02 ^j^
**Sinapic Acid**	0.11 ^d^	0.14 ^b^	0.05 ^e^	0.13 ^c^	0.24 ^a^	0.13 ^c^	0.00	0.00	0.00	0.00	0.00	0.00
**Ferulic Acid**	0.28 ^e^	0.35 ^d^	0.18 ^f^	0.47 ^c^	0.79 ^b^	1.70 ^a^	0.01 ^l^	0.05 ^i^	0.02 ^k^	0.17 ^g^	0.08 ^h^	0.03 ^j^
**Kaempferol 3-*O*-Glucoside**	0.84 ^f^	0.11 ^i^	0.16 ^h^	0.83 ^f,g^	0.08 ^j^	0.17 ^h^	2.17 ^a^	1.03 ^e^	0.82 ^g^	1.54 ^b^	1.34 ^c^	1.17 ^d^
**Quercetin**	3.47 ^g^	10.12 ^d^	2.88 ^h^	19.25 ^c^	53.69 ^a^	26.51 ^b^	3.54 ^f^	2.46 ^k^	2.45 ^k^	3.99 ^e^	2.57 ^j^	2.77 ^i^
**Cinnamic Acid**	0.04 ^h^	0.03 ^i^	0.05 ^g^	0.04 ^h^	0.03 ^i^	0.07 ^f^	0.45 ^c^	0.80 ^b^	0.34 ^d^	1.36 ^a^	0.26 ^e^	0.34 ^d^
**Naringenin**	0.28 ^a^	0.27 ^b^	0.24 ^d^	0.25 ^c^	0.28 ^a^	0.25 ^c^	0.00	0.00	0.00	0.00	0.00	0.00
**Kaempferol**	0.00	0.00	0.00	0.73 ^d^	0.68 ^f^	0.61 ^g^	0.80 ^b^	0.79 ^b^	0.77 ^c^	1.74 ^a^	0.70 ^e^	0.71 ^e^
***cis, trans*-Abscisic Acid**	1.40 ^a^	0.70 ^d^	0.97 ^c^	1.04 ^b^	0.25 ^f^	0.65 ^e^	0.04 ^k^	0.13 ^j^	0.19 ^h^	0.13 ^j^	0.24 ^g^	0.16 ^i^
**TPC**	2.27 ^f^	4.08 ^c^	2.69 ^e^	4.38 ^b^	6.26 ^a^	3.30 ^d^	1.70 ^h^	1.58 ^i^	1.47 ^j^	2.27 ^f^	1.18 ^k^	2.04 ^g^
**RSA**	18.94 ^g^	25.36 ^c^	18.31 ^h^	23.49 ^e^	33.83 ^a^	33.03 ^b^	16.69 ^k^	18.15 ^i^	17.71 ^j^	24.05 ^d^	16.32 ^l^	23.35 ^f^
**TAC**	0.62 ^f^	1.80 ^b^	0.82 ^e^	1.05 ^d^	2.86 ^a^	1.63 ^c^	0.16 ^k^	0.23 ^i^	0.22 ^i^	0.37 ^g^	0.19 ^j^	0.33 ^h^

* The relative standard deviation (RSD) values were less than 5% for all the analysis. ** Different letter in the same row denotes a significant difference among cultivars/cultivation systems according to Tukey′s test, *p* < 0.05.

**Table 2:** Contents of quantified phenolics (mg/kg) *, TPC (g GAE/kg) * and RSA (mmol TE/kg) * in leaves from three blueberry cultivars and three strawberry cultivars, West Serbia in 2014.

Compound Name	Blueberry Leaves						Strawberry Leaves					
	Integrated			Organic			Integrated			Organic		
	Bluecrop	Duke	Nui	Bluecrop	Duke	Nui	Alba	Favette	Clery	Alba	Favette	Clery
**Gallocatechin**	21.29 ^b,^**	15.63 ^d^	34.60 ^a^	17.79 ^c^	14.11 ^e^	11.17 ^f^	0.00	0.00	0.00	0.00	0.00	0.00
**Aesculin**	3.24 ^c^	2.57 ^d^	2.54 ^d^	3.41 ^b^	2.40 ^e^	3.70 ^a^	0.94 ^f^	0.64 ^i^	0.75 ^h^	0.83 ^g^	0.17 ^k^	0.48 ^j^
**5-*O*-Caffeoylquinic Acid**	1826.07 ^c^	1359.64 ^f^	1617.33 ^d^	2108.73 ^b^	1485.93 ^e^	2380.63 ^a^	176.49 ^g^	176.64 ^g^	152.88 ^h^	92.22 ^i^	5.82 ^k^	9.11 ^j^
**Catechin**	7.62 ^g^	6.00 ^i^	13.50 ^d^	7.57 ^g^	7.08 ^h^	4.51 ^j^	0.31 ^k^	9.47 ^f^	11.67 ^e^	14.69 ^c^	30.36 ^b^	31.86 ^a^
***p*-Hydroxybenzoic Acid**	4.72 ^d^	4.25 ^e^	3.93 ^f^	5.86 ^a^	4.80 ^c^	4.90 ^b^	1.68 ^j^	1.96 ^i^	1.52 ^l^	2.10 ^h^	1.60 ^k^	2.20 ^g^
**Caffeic Acid**	55.51 ^b^	26.40 ^f^	41.76 ^e^	51.16 ^c^	42.94 ^d^	90.68 ^a^	5.21 ^l^	13.25 ^g^	6.76 ^k^	8.11 ^j^	12.23 ^h^	12.02 ^i^
**Syringic Acid**	0.00	0.00	0.00	0.00	0.00	0.00	0.48 ^b^	0.30 ^d^	0.30 ^d^	0.24 ^e^	0.63 ^a^	0.33 ^c^
**Apigenin 8-*C*-Glucoside**	10.06 ^c^	7.48 ^f^	8.03 ^e^	10.62 ^b^	9.68 ^d^	13.17 ^a^	0.00	0.00	0.00	0.00	0.00	0.00
**Quercetin 3-*O*-Rutinoside**	13.19 ^d^	6.63 ^f^	26.37 ^b^	12.73 ^e^	14.22 ^c^	27.01 ^a^	0.00	0.00	0.00	0.00	0.00	0.00
***p*-Coumaric Acid**	2.96 ^i^	0.16 ^k^	3.05 ^h^	3.76 ^g^	2.40 ^j^	3.81 ^g^	5.15 ^f^	10.36 ^a^	6.09 ^e^	6.49 ^d^	9.47 ^b^	8.64 ^c^
**Quercetin 3-*O*-Galactoside**	124.77 ^b^	90.72 ^f^	99.65 ^e^	125.67 ^c^	118.57 ^d^	130.20 ^a^	4.56 ^k^	8.25 ^j^	9.65 ^h^	9.36 ^i^	12.53 ^g^	13.35 ^g^
**Vanillic Acid**	0.60 ^h^	0.60 ^h^	1.32 ^d^	1.92 ^b^	1.14 ^f^	3.51 ^a^	1.06 ^g^	1.20 ^e^	0.61 ^h^	1.51 ^c^	0.88 ^i^	1.31 ^d^
**Sinapic Acid**	0.48 ^g^	0.16 ^i^	0.30 ^h^	0.32 ^h^	0.14 ^j^	0.67 ^f^	3.23 ^e^	27.47 ^a^	15.56 ^b^	14.47 ^c^	0.04 ^k^	5.64 ^d^
**Ferulic Acid**	0.99 ^e^	0.48 ^j^	0.55 ^i^	0.80 ^g^	0.46 ^k^	1.39 ^c^	1.29 ^d^	1.54 ^b^	0.87 ^f^	0.81 ^g^	0.73 ^h^	2.44 ^a^
**Kaempferol 3-*O*-Glucoside**	38.62 ^b^	4.09 ^f^	11.10 ^d^	41.07 ^a^	6.77 ^e^	12.65 ^c^	1.20 ^k^	1.29 ^j^	1.69 ^i^	3.28 ^g^	2.41 ^h^	0.52 ^l^
**Quercetin**	151.49 ^c^	165.65 ^b^	89.51 ^e^	83.57 ^f^	145.90 ^d^	214.72 ^a^	36.09 ^g^	20.82 ^j^	20.06 ^k^	21.67 ^i^	32.35 ^h^	14.14 ^l^
**Cinnamic Acid**	0.35 ^g^	0.26 ^i^	0.28 ^h^	0.09 ^j^	0.27 ^h,i^	0.56 ^e,f^	0.57 ^e^	1.04 ^b^	0.55 ^f^	0.66 ^d^	1.18 ^a^	0.88 ^c^
**Naringenin**	0.71 ^e^	0.52 ^j^	0.66 ^g^	0.65 ^h^	0.57 ^i^	0.85 ^b^	0.68 ^f^	0.83 ^c^	0.72 ^e^	0.65 ^h^	0.91 ^a^	0.75 ^d^
**Kaempferol**	14.44 ^a^	5.22 ^d^	4.93 ^e^	9.01 ^b^	4.00 ^i^	8.25 ^c^	4.08 ^g^	2.83 ^k^	3.13 ^j^	4.43 ^f^	4.04 ^h^	2.41 ^l^
**Pinocembrin**	0.00	0.00	0.00	0.00	0.00	0.00	0.31 ^b,c^	0.33 ^a^	0.32 ^b^	0.30 ^c,d^	0.33 ^a^	0.29 ^d^
**TPC**	54.56 ^e^	46.96 ^g^	55.75 ^d^	81.06 ^b^	55.42 ^d^	77.65 ^c^	49.89 ^f^	45.75 ^h^	44.83 ^i^	38.22 ^k^	41.29 ^j^	82.25 ^a^
**RSA**	456.50 ^i^	336.19 ^l^	423.25 ^j^	493.62 ^h^	341.53 ^k^	679.94 ^g^	802.29 ^f^	1880.32 ^b^	1483.77 ^c^	1225.43 ^d^	2237.31 ^a^	951.24 ^e^

* The relative standard deviation (RSD) values were less than 5% for all the analysis. ** Different letter in the same row denotes a significant difference among cultivars/cultivation systems according to Tukey′s test, *p* < 0.05.

**Table 3:** Ultra-high performance liquid chromatography-MS ^4^ (UHPLC-MS ^4^) data about identification of main compounds in organic and integral strawberry and blueberry extracts.

No	*t*_R_, min	Compound Name	Molecular Formula [M − H]^−^	Calculated Mass [M − H]^−^	Exact Mass [M − H]^−^	Δ ppm	MS ^2^ Fragments, (% Base Peak)	MS ^3^ Fragments, (% Base Peak)	MS ^4^ Fragments, (% Base Peak)
**1**	2.94	**Gallic Acid Hexoside Isomer 1**	C_13_H_15_O_10_^–^	331.06707	331.06616	2.75	294(10), **169**(100), 125(5)	**125**(100)	107(100), 81(10)
**2**	4.17	**Dihydroxybenzoic Acid hexoside Isomer 1**	C_13_H_15_O_9_^–^	315.07216	315.07104	3.55	**153**(100), 152(50), 109(15), 108(10)	**109**(100)	123(25), 109(10), 85(10), 81(100)
**3**	4.31	**Gallic Acid Hexoside Isomer 2**	C_13_H_15_O_10_^–^	331.06707	331.06622	2.57	**169**(100), 125(5)	**125**(100)	110(10), 97(30), 81(100), 53(30)
**4**	4.44	**Prodelphinidin Dimer B Type**	C_30_H_25_O_13_^–^	593.13006	593.12878	2.16	467(15), **425**(100), 407(30), 289(20)	**407**(100), 381(5), 273(10)	389(30), 297(30), 285(100), 243(70)
**5**	4.53	**Caffeoyltartaric Acid**	C_13_H_11_O_9_^–^	311.04031	311.03986	1.45	179(50), 177(10), **149**(100)	131(60), 103(90), **87**(100), 59(20)	59(100)
**6**	4.54	**Chlorogenic Acid Hexoside Isomer 1**	C_22_H_27_O_14_^–^	515.14008	515.14001	0.14	**353**(100), 341(5), 323(10), 191(90), 179(5)	**191**(100), 179(10)	173(65), 127(80), 111(30), 85(100)
**7**	4.59	**Gallocatechin** *	C_15_H_13_O_7_^−^	305.06668	305.06537	4.29	261(50), 221(70), 219(70), **179**(100), 165(35)	**164**(100), 151(40), 135(30)	120(100), 108(20)
**8**	4.64	**Dihydroxybenzoic Acid Hexosyl-Pentoside**	C_18_H_23_O_13_^–^	447.11441	447.11353	1.97	**315**(100), 285(10), 153(10)	**153**(100), 123(10)	123(100)
**9**	4.71	**Gallic Acid Hexoside Isomer 3**	C_13_H_15_O_10_^–^	331.06707	331.06610	2.93	**313**(100), 211(10), 169(30), 168(80), 150(10), 125(25)	193(50), **151**(100), 125(80)	123(100), 107(90), 95(65)
**10**	4.72	**Chlorogenic Acid Hexoside Isomer 2**	C_22_H_27_O_14_^–^	515.14008	515.13928	1.55	353(40), **341**(100), 335(30), 323(10), 191(15), 179(45)	**179**(100), 135(10)	135(100)
**11**	4.83	**Caffeic Acid Hexoside Isomer 1**	C_15_H_17_O_9_^–^	341.08781	341.08685	2.81	191(10), **179**(100), 135(10)	**135**(100)	135(100), 107(50)
**12**	4.84	**Dihydroxybenzoic Acid Pentoside**	C_12_H_13_O_8_^–^	285.06159	285.06094	2.28	**153**(100), 152(25), 109(5), 108(5)	**109**(100)	81(100)
**13**	4.90	**3-*O*-Caffeoylquinic Acid Isomer 1**	C_16_H_17_O_9_^–^	353.08781	353.08673	3.06	**191**(100), 179(30), 135(10)	173(75), **127**(100), 111(40), 93(60), 85(90)	109(30), 99(40), 85(100)
**14**	5.02	**Hydroxybenzoic Acid Hexoside**	C_13_H_15_O_8_^–^	299.07724	299.07693	1.04	**137**(100)	**93**(100)	–
**15**	5.09	**3-*O*-Caffeoylquinic Acid Isomer 2**	C_16_H_17_O_9_^–^	353.08781	353.08652	3.65	**191**(100), 179(30), 135(10)	173(75), **127**(100), 111(40), 93(60), 85(90)	109(30), 99(40), 85(100)
**16**	5.10	**Procyanidin Dimer B Type Isomer 1**	C_30_H_25_O_12_^–^	577.13515	577.13434	1.40	559(10), 451(30), **425**(100), 407(40), 289(20), 287(10)	**407**(100), 381(5), 287(5), 273(10)	389(30), 297(30), 285(100), 281(90)
**17**	5.12	**Aesculin** *	C_15_H_15_O_9_^–^	339.07216	339.07114	3.01	**177**(100)	177(5), 149(10), **133**(100), 105(10), 89(5)	89(100)
**18**	5.28	**Caffeic Acid Hexoside Isomer 2**	C_15_H_17_O_9_^–^	341.08781	341.08664	3.43	**179**(100), 135(10)	**135**(100)	107(100), 79(20)
**19**	5.31	**Coumaric Acid Hexoside Isomer 1**	C_15_H_17_O_8_^–^	325.09289	325.09171	3.63	**163**(100), 119(10)	**119**(100)	–
**20**	5.33	**Procyanidin Dimer B Type Isomer 2**	C_30_H_25_O_12_^–^	577.13515	577.13409	1.84	559(5), 451(20), **425**(100), 407(35), 289(20), 287(10)	**407**(100), 381(10), 273(10)	389(40), 297(40), 285(100), 243(75)
**21**	5.35	**5-*O*-Caffeoylquinic Acid** *	C_16_H_17_O_9_^–^	353.08781	353.08616	4.67	**191**(100), 179(5)	173(75), **127**(100), 111(40), 93(60), 85(90)	109(40), 99(50), 85(100)
**22**	5.37	**Epigallocatechin** *	C_15_H_13_O_7_^–^	305.06668	305.06589	2.59	287(10), 261(40), 247(20), 221(90), 219(80), **179**(100)	**164**(100), 151(40), 135(30)	120(100), 108(20)
**23**	5.44	**Dihydroxybenzoic Acid Hexoside Isomer 2**	C_13_H_15_O_9_^–^	315.07216	315.07123	2.95	**153**(100), 135(10), 109(10)	**135**(100), 109(50)	91(100)
**24**	5.45	**Quercetin 3-*O*-Hexoside-7-*O*-hexuronide**	C_27_H_27_O_18_^–^	639.12029	639.11963	1.03	**463**(100), 301(20)	343(5), **301**(100)	179(70), 151(100), 107(10)
**25**	5.47	**Catechin** *	C_15_H_13_O_6_^–^	289.07176	289.07068	3.74	271(5), **245**(100), 205(40), 179(15), 125(5)	227(30), **203**(100), 187(25), 175(10), 161(20)	188(70), 185(20), 175(100), 161(40), 157(10)
**26**	5.48	***p*-Hydroxybenzoic Acid** *	C_7_H_5_O_3_^–^	137.02442	137.02420	1.61	109(10), **93**(100)	**93**(10)	–
**27**	5.57	**Coumaric Acid Hexoside Isomer 2**	C_15_H_17_O_8_^–^	325.09289	325.09128	4.95	289(20), 265(20), 235(10), 187(40), 163(80), **145**(100)	**117**(100)	–
**28**	5.60	**4-*O*-Caffeoylquinic Acid**	C_16_H_17_O_9_^–^	353.08781	353.08688	2.63	191(60), 179(75), **173**(100), 135(15)	115(20), 111(50), **93**(100), 71(20)	–
**29**	5.61	**Procyanidin Dimer B Type Isomer 3**	C_30_H_25_O_12_^–^	577.13515	577.13312	3.52	559(10), 451(20), **425**(100), 407(40), 289(20), 287(10)	**407**(100), 381(5), 273(10)	389(30), 297(30), 285(100), 243(75)
**30**	5.80	**Methyl 3-caffeoylquinate**	C_17_H_19_O_9_^–^	367.10346	367.10251	2.59	193(20), 179(5), **161**(100), 135(10)	**133**(100)	77(100)
**31**	5.84	**Caffeic Acid** *	C_9_H_7_O_4_^–^	179.03498	179.03444	3.02	**135**(100)	135(60), 117(15), **107**(100), 91(55), 79(15)	–
**32**	5.92	**Epicatechin** *	C_15_H_13_O_6_^–^	289.07176	289.07104	2.49	271(5), **245**(100), 205(40), 179(15), 125(5)	227(35), **203**(100), 187(30), 175(15), 161(25)	188(60), 185(20), 175(100), 161(35), 157(15)
**33**	5.92	**5-Caffeoylquinic acid Isomer**	C_16_H_17_O_9_^–^	353.08781	353.08624	4.45	**191**(100), 179(5)	173(75), **127**(100), 111(40), 93(60), 85(90)	109(40), 99(50), 85(100)
**34**	6.00	**Syringic Acid** *	C_9_H_9_O_5_^−^	197.04555	197.04477	3.96	**183**(100), 153(40), 138(10)	**167**(100), 138(10), 123(5)	–
**35**	6.01	**Caffeoylshikimic Acid**	C_16_H_15_O_8_^–^	335.07724	335.07587	4.09	**179**(100), 135(25)	**135**(100)	107(100)
**36**	6.06	**Myricetin 3-*O*-rutinoside**	C_27_H_29_O_17_^–^	625.14102	625.14014	1.41	607(10), 359(5), 329(5), 317(65), **316**(100), 287(10)	287(30), **271**(100), 179(30), 151(10)	271(10), 243(100), 227(40), 215(15)
**37**	6.14	**Quercetin 3-*O*-Hexosyl-hexuronide**	C_27_H_27_O_18_^–^	639.12029	639.11865	2.57	337(10), **301**(100)	273(20), 257(20), **179**(100), 151(75)	151(100)
**38**	6.18	**Myricetin 3-*O*-hexoside**	C_21_H_19_O_13_^–^	479.08311	479.08176	2.82	**317**(100), 316(80)	273(60), **179**(100), 151(40)	151(100)
**39**	6.22	**Methyl 4-caffeoylquinate**	C_17_H_19_O_9_^–^	367.10346	367.10211	3.68	193(5), 179(5), **161**(100), 135(30)	**133**(100)	105(100)
**40**	6.26	**Ellagic Acid Pentoside**	C_19_H_13_O_12_^–^	433.04125	433.04047	1.80	**301**(100), 300(80)	301(95), 284(25), **257**(100), 229(70), 222(15)	229(70), 213(30), 201(15), 185(100)
**41**	6.29	**Methyl 3-*p*-coumaroylquinate**	C_17_H_19_O_8_^–^	351.10854	351.10767	2.48	163(5), **145**(100), 119(10), 117(10)	**117**(100)	–
**42**	6.40	**Ellagic Acid Rhamnoside**	C_20_H_15_O_12_^–^	447.05690	447.05576	2.55	301(50), **300**(100)	**300**(100), 284(15), 271(20), 257(30), 244(30)	216(100), 200(40), 188(15), 172(20)
**43**	6.44	**Apigenin 8-*C*-glucoside** *	C_21_H_19_O_10_^−^	431.09837	431.09720	2.71	341(20), **311**(100)	**283**(100)	283(50), 239(100), 224(40), 197(50), 183(60)
**44**	6.45	**Methyl 5-caffeoylquinate isomer 1**	C_17_H_19_O_9_^–^	367.10346	367.10190	4.25	191(20), **179**(100), 161(10), 135(50)	**135**(100)	135(60), 107(100), 91(25), 79(20)
**45**	6.46	**Coumaric Acid Hexoside Isomer 3**	C_15_H_17_O_8_^–^	325.09289	325.09180	3.35	289(10), 265(10), **163**(100), 161(50), 119(60), 101(20)	**91**(100)	–
**46**	6.47	**Quercetin 3-*O*-rutinoside** *	C_27_H_29_O_16_^–^	609.14611	609.14496	1.89	343(5), **301**(100), 300(30), 271(10), 255(5)	273(25), 257(20), **179**(100), 151(75)	151(100)
**47**	6.50	**Myricetin 3-*O*-pentoside**	C_20_H_17_O_12_^–^	449.07255	449.07169	1.92	387(5), 317(35), **316**(100)	287(30), **271**(100), 179(30), 151(10)	271(10), 243(100), 227(40), 215(15)
**48**	6.65	***p*-Coumaric acid** *	C_9_H_7_O_3_^–^	163.04007	163.03932	4.60	**119**(100)	119(60), 101(20), 93(25), **91**(100), 72(10)	–
**49**	6.67	**Quercetin 3-*O*-galactoside**	C_21_H_19_O_12_^–^	463.08820	463.08719	2.18	**301**(100), 300(30)	273(25), 257(20), **179**(100), 151(75)	151(100)
**50**	6.70	**Methyl 5-caffeoylquinate Isomer 2**	C_17_H_19_O_9_^–^	367.10346	367.10269	2.10	191(20), **179**(100), 161(10), 135(50)	**135**(100)	109(100), 107(70)
**51**	6.75	**Ellagic Acid**	C_14_H_5_O_8_^–^	300.99899	300.99805	3.12	284(40), 271(60), **257**(100), 229(85), 185(40)	**229**(100), 213(20), 185(85)	201(100), 185(95), 157(30), 145(20)
**52**	6.82	**Kaempferol 7-*O*-rutinoside**	C_27_H_29_O_15_^–^	593.15119	593.14972	2.48	**285**(100)	267(40), **257**(100), 241(30), 229(40), 213(30)	255(10), 239(30), 229(100), 163(40)
**53**	6.85	**Quercetin 3-*O*-rhamnosyl-hexuronide**	C_27_H_27_O_17_^–^	623.12537	623.12341	3.15	605(15), 491(10), 475(5), 315(40), 301(60), **300**(100)	**271**(100), 255(60), 179(10), 151(10)	243(100), 227(80), 215(20), 199(20)
**54**	6.87	**Vanillic Acid** *	C_8_H_7_O_4_^–^	167.03498	167.03419	4.73	153(10), 152(80), 124(10), **123**(100), 108(20)	**108**(100)	79(100)
**55**	6.89	**Isorhamnetin 3-*O*-rutinoside**	C_28_H_31_O_16_^–^	623.16176	623.16010	2.66	**315**(100), 300(20), 271(10), 255(5)	**300**(100), 287(5), 272(5)	271(100), 255(50), 151(5)
**56**	6.97	**Quercetin 3-*O*-pentoside**	C_20_H_17_O_11_^–^	433.07763	433.07669	2.17	343(5), 301(80), **300**(100)	**271**(100), 255(60), 179(10), 151(10)	243(100), 227(80), 215(20), 199(20)
**57**	6.99	**Sinapic Acid** *	C_11_H_11_O_5_^−^	223.06120	223.06058	2.78	**208**(100), 179(30), 164(20)	193(10), **164**(100), 149(15), 135(5)	149(100), 135(35)
**58**	7.02	**Ferulic Acid** *	C_10_H_9_O_4_^–^	193.05063	193.04990	3.78	178(70), **149**(100), 134(40)	**134**(100)	–
**59**	7.05	**Methyl 5-*p*-coumaroylquinate Isomer 1**	C_17_H_19_O_8_^–^	351.10854	351.10773	2.31	**163**(100), 145(5), 119(15)	**119**(100)	–
**60**	7.08	**Kaempferol 3-*O*-glucoside** *	C_21_H_19_O_11_^–^	447.09329	447.09244	1.90	327(20), 285(80), **284**(100), 255(10)	**255**(100), 227(10)	227(100), 211(60)
**61**	7.12	**Syringetin 3-*O*-hexoside**	C_23_H_23_O_13_^–^	507.11441	507.11292	2.94	479(10), 387(20), 345(80), **344**(100), 299(15)	330(90), **316**(100), 301(90), 287(10), 273(70)	301(100), 300(20), 287(10), 273(60)
**62**	7.16	**Isorhamnetin 3-*O*-hexoside**	C_22_H_21_O_12_^–^	477.10385	477.10321	1.34	357(20), 315(50), **314**(100), 300(5), 299(5), 285(10)	300(30), **285**(100), 271(75), 257(10), 243(25)	270(100)
**63**	7.20	**Quercetin 3-*O*-acetyl-hexoside Isomer 1**	C_23_H_21_O_13_^–^	505.09876	505.09756	2.38	463(20), 343(20), **301**(100), 300(60), 299(50)	273(20), 257(20), **179**(100), 151(75)	151(100)
**64**	7.24	**Isorhamnetin 3-*O*-hexuronide**	C_22_H_19_O_13_^–^	491.08311	491.08221	1.83	473(10), 315(70), **301**(100), 300(60)	283(15), 272(20), 256(10), **179**(100), 151(75)	151(100)
**65**	7.29	**Dicaffeoylquinic Acid Isomer 1**	C_25_H_23_O_12_^–^	515.11950	515.11789	3.13	**353**(100)	**191**(100), 179(40), 135(10)	173(100), 127(50), 111(40), 85(70)
**66**	7.31	**Methyl 5-*p*-coumaroylquinate Isomer 2**	C_17_H_19_O_8_^–^	351.10854	351.10773	2.31	**163**(100), 145(5), 119(15)	**119**(100)	–
**67**	7.38	**Quercetin 3-*O*-acetyl-hexoside Isomer 2**	C_23_H_21_O_13_^–^	505.09876	505.09744	2.61	463(30), 445(30), 343(5), **301**(100), 300(90), 299(10)	272(40), 256(25), **179**(100), 151(75)	151(100)
**68**	7.44	**Quercetin 3-*O*-methyl-malonyl-hexoside**	C_25_H_23_O_15_^–^	563.10424	563.10309	2.04	**531**(100), 463(80)	**463**(100)	343(5), 301(100), 300(30)
**69**	7.45	**Quercetin 7-*O*-hexuronide**	C_21_H_17_O_13_^–^	477.06692	477.06580	2.35	**301**(100)	273(20), 257(20), **179**(100), 151(75)	151(100)
**70**	7.46	**Isorhamnetin 3-*O*-pentoside**	C_21_H_19_O_11_^–^	447.09329	447.09265	1.43	357(10), 315(30), **314**(100), 285(5), 271(5)	300(10), **285**(100), 271(70), 257(10), 243(20)	270(100)
**71**	7.49	**Quercetin 3-*O*-malonyl-hexoside**	C_24_H_21_O_15_^–^	549.08859	549.08752	1.95	**505**(100)	463(30), **301**(100), 300(50)	273(15), 257(15), 179(100), 151(85)
**72**	7.54	**Dicaffeoylquinic Acid Isomer 2**	C_25_H_23_O_12_^–^	515.11950	515.11865	1.65	**353**(100)	**191**(100), 179(40), 173(20), 135(10)	173(60), 127(100), 111(40), 85(80)
**73**	7.60	**Kaempferol 7-*O*-hexuronide**	C_21_H_17_O_12_^–^	461.07200	461.07141	1.28	**285**(100)	267(40), **257**(100), 241(30), 229(50), 213(25)	255(10), 239(30), 229(100), 163(60)
**74**	7.63	**Isorhamnetin 3-*O*-malonyl-rutinoside**	C_31_H_33_O_19_^–^	709.16160	709.16040	1.69	666(30), **665**(100)	623(15), **315**(100), 300(20), 271(15), 255(10)	300(100), 287(10), 272(10), 256(5)
**75**	7.64	**Myricetin**	C_15_H_9_O_8_^−^	317.03029	317.02927	3.22	287(30), 271(15), 193(10), **179**(100),1151(45)	**151**(100)	107(100), 83(15)
**76**	7.70	**Methyl 3,4-dicaffeoylquinate**	C_26_H_25_O_12_^–^	529.13515	529.13397	2.23	**367**(100), 161(10)	335(5), 193(10), 179(5), **161**(100), 135(20)	133(100)
**77**	7.73	**Kaempferol 3-*O*-hexuronide methyl Ether**	C_22_H_19_O_12_^−^	475.08820	475.08701	2.50	327(10), 301(10), 285(70), **284**(100), 255(35), 227(5)	**255**(100), 227(10)	227(100), 211(60)
**78**	8.00	**Kaempferol 3-*O*-malonyl-hexoside**	C_24_H_21_O_14_^–^	533.09368	533.09229	2.61	**489**(100)	**285**(100)	267(40), 257(100), 241(30), 229(50)
**79**	8.02	**Methyl Caffeate**	C_10_H_9_O_4_^–^	193.05063	193.05019	2.28	178(30), **161**(100), 134(70), 111(10)	**133**(100)	–
**80**	8.04	**Methyl 3,5-dicaffeoylquinate**	C_26_H_25_O_12_^–^	529.13515	529.13391	2.34	**367**(100), 349(10), 179(10), 161(10)	335(5), 193(10), 191(25), **179**(100), 161(80)	135(100)
**81**	8.16	**Feruloyl-coumaroylquinic acid isomer 1**	C_26_H_25_O_11_^–^	513.14024	513.13953	1.38	**367**(100), 351(5), 161(10)	335(5), 193(10), 179(5), **161**(100), 135(30)	133(100)
**82**	8.29	**Methyl 4,5-dicaffeoylquinate**	C_26_H_25_O_12_^–^	529.13515	529.13385	2.46	**367**(100), 349(10), 179(15), 161(10)	335(10), 193(10), 191(20), **179**(100), 161(80)	135(100)
**83**	8.30	**Kaempferol3-*O*-*p*-coumaroyl-hexoside**	C_30_H_25_O_13_^–^	593.13006	593.12769	4.00	447(15), 307(10), **285**(100)	**257**(100), 241(50), 229(35), 213(40), 151(90)	256(10), 239(25), 229(100), 213(20)
**84**	8.49	***cis, trans*-Abscisic acid** *	C_15_H_19_O_4_^−^	263.12888	263.12839	1.86	219(15), **153**(100), 151(5)	**138**(100), 109(10), 97(15)	122(100)
**85**	8.53	**Feruloyl-coumaroylquinic Acid Isomer 2**	C_26_H_25_O_11_^–^	513.14024	513.13904	2.34	367(70), **349**(100), 179(5), 161(10)	305(20), 179(10), **161**(100), 133(15)	133(100)
**86**	8.62	**Quercetin** *	C_15_H_9_O_7_^−^	301.03538	301.03442	3.19	271(50), 255(20), **179**(100), 151(80), 107(5)	**151**(100)	107(100), 83(10)
**87**	8.78	**Feruloyl-coumaroylquinic Acid Isomer 3**	C_26_H_25_O_11_^–^	513.14024	513.14008	0.31	367(70), **349**(100), 337(10), 179(5), 163(15), 161(10)	305(10), 193(20), 173(15), **161**(100), 133(10)	133(100)
**88**	9.03	**Cinnamic Acid** *	C_9_H_7_O_2_^−^	147.04515	147.04463	3.54	104(10), **103**(100), 87(10)	**119**(100)	–
**89**	9.32	**Naringenin** *	C_15_H_11_O_5_^−^	271.06120	271.06039	2.99	177(10), **151**(100)	**107**(100)	65(100)
**90**	9.51	**Kaempferol** *	C_15_H_9_O_6_^−^	285.04046	285.03909	4.81	**255**(100), 227(10)	**211**(100), 195(5), 167(15)	211(40), 137(100)
**91**	9.57	**Syringetin**	C_17_H_13_O_8_^–^	345.06159	345.06036	3.56	**330**(100), 315(10), 300(5)	**315**(100)	287(100), 271(40), 259(25), 243(15)
**92**	9.69	**Isorhamnetin**	C_16_H_11_O_7_^–^	315.05103	315.04985	3.75	301(20), **300**(100)	283(40), 271(80), 255(30), 227(30), **151**(100)	107(100), 83(15)
**93**	11.46	**Pinocembrin** *	C_15_H_11_O_4_^−^	255.06628	255.06580	1.88	**213**(100), 187(15), 151(30), 145(10), 107(5)	**185**(100), 169(20), 145(20)	185(10), 157(15), 143(100), 141(50)

* Conformed using available standards; The other compounds were identified using HRMS and MS ^n^ data available in literature; *t*_R_—retention time; Δ ppm—Mean mass accuracy. “–“ Stands for not detected fragment.

## Supplementary Materials

The following are available online, Figure S1: Proposed structure and fragmentation pathway of compound **37**, Table S1: Presence of each identified compound in integrated and organic strawberry and blueberry fruit samples, Table S2: Presence of each identified compound in integrated and organic strawberry and blueberry leaf samples.
