# Supplementary material for: Chemometric Characterization of Strawberries and Blueberries according to Their Phenolic Profile: Combined Effect of Cultivar and Cultivation System
Source: Molecules. 2019 Nov 26;24(23):4310. doi: 10.3390/molecules24234310 (PMC6930459; doi:10.3390/molecules24234310)
Supplement: Supplementary file 1 [file molecules-24-04310-s001.pdf]

Supplementary material

Article

Chemometric characterization of strawberries and blueberries according to their phenolic profile: combined effect of cultivar and cultivation system

Milica Fotirić Akšić [1,*], Dragana Dabić Zagorac [2], Milica Sredojević [2], Jasminka Milivojević[1], Uroš Gašić [3], Mekjell Meland [4], and Maja Natić [5]

[1] University of Belgrade –Faculty of Agriculture, 11080 Belgrade, Serbia; fotiric@agrif.bg.ac.rs (M.F.A.); jasminka@agrif.bg.ac.rs (J.M.)

[2] Innovation Center, University of Belgrade - Faculty of Chemistry, P.O. Box 51, 11158 Belgrade, Serbia; ddabic@chem.bg.ac.rs (D.D.Z.); pantelicm@chem.bg.ac.rs (M.S.)

[3] Institute for Biological Research "Siniša Stanković"–National Institute of Republic of Serbia, University of Belgrade, 11060 Belgrade, Serbia; uros.gasic@ibiss.bg.ac.rs (U.G.)

[4] Norwegian Institute of Bioeconomy Research - NIBIO Ullensvang, Lofthus, Norway; mekjell.meland@nibio.no (M.M.)

[5] University of Belgrade - Faculty of Chemistry, P.O. Box 51, 11158 Belgrade, Serbia; mmandic@chem.bg.ac.rs (M.N.)

* Correspondence: fotiric@agrif.bg.ac.rs; Tel.: +381642612710

Content

Table S1. Presence of each identified compound in integrated and organic strawberry and blueberry fruit samples.

Table S2. Presence of each identified compound in integrated and organic strawberry and blueberry leaf samples.

Figure S1. Proposed fragmentation pathway of compound **37**.

Table S1. Presence of each identified compound in integrated and organic strawberry and blueberry fruit samples.

No	Compound name	Blueberry-fruit						Strawberry-fruit					
		Integrated			Organic			Integrated			Organic		
		Bluecrop	Duke	Nui	Bluecrop	Duke	Nui	Alba	Favette	Clery	Alba	Favette	Clery
1	Gallic acid hexoside isomer 1	–	–	–	–	–	–	–	–	–	–	–	–
2	Dihydroxybenzoic acid hexoside isomer 1	+	+	+	+	+	+	+	+	+	+	+	+
3	Gallic acid hexoside isomer 2	+	+	+	+	+	+	+	+	+	+	+	+
4	Prodelphinidin dimer B type	+	+	+	+	+	+	–	–	–	–	–	–
5	Caffeoyltartaric acid	–	–	–	–	–	–	–	–	–	–	–	–
6	Chlorogenic acid hexoside isomer 1	–	–	–	–	–	–	–	–	–	–	–	–
7	Gallocatechin	–	–	–	–	–	–	–	–	–	–	–	–
8	Dihydroxybenzoic acid hexosyl-pentoside	–	–	–	–	–	–	–	–	–	–	–	–
9	Gallic acid hexoside isomer 3	–	–	–	–	–	–	+	+	+	+	+	+
10	Chlorogenic acid hexoside isomer 2	–	–	–	–	–	–	–	–	–	–	–	–
11	Caffeic acid hexoside isomer 1	+	+	+	+	+	+	–	–	–	–	–	–
12	Dihydroxybenzoic acid pentoside	+	+	+	+	+	+	+	+	+	+	+	+
13	3-*O*-Caffeoylquinic acid isomer 1	–	–	–	–	–	–	–	–	–	–	–	–
14	Hydroxybenzoic acid hexoside	–	–	–	–	–	–	+	+	+	+	+	+
15	3-*O*-Caffeoylquinic acid isomer 2	–	–	–	–	–	–	–	–	–	–	–	–
16	Procyanidin dimer B type isomer 1	+	+	+	+	+	+	+	+	+	+	+	+
17	Aesculin	–	–	–	+	+	+	–	–	–	–	–	–
18	Caffeic acid hexoside isomer 2	+	+	+	+	+	+	+	+	+	+	+	+
19	Coumaric acid hexoside isomer 1	+	+	+	+	+	+	–	–	–	–	–	–
20	Procyanidin dimer B type isomer 2	+	+	+	+	+	+	+	+	+	+	+	+
21	5-*O*-Caffeoylquinic acid	+	+	+	+	+	+	–	–	–	–	–	–
22	Epigallocatechin	+	+	+	+	+	+	–	–	–	–	–	–
23	Dihydroxybenzoic acid hexoside isomer 2	–	–	–	–	–	–	+	+	+	+	+	+
24	Quercetin 3-*O*-hexosyl-hexuronide	–	–	–	–	–	–	–	–	–	–	–	–
25	Catechin	+	+	+	+	+	+	+	+	+	+	+	+
26	*p*-Hydroxybenzoic acid	+	+	+	+	+	+	+	+	+	+	+	+
27	Coumaric acid hexoside isomer 2	+	+	+	+	+	+	+	+	+	+	+	+
28	4-*O*-Caffeoylquinic acid	–	–	–	–	–	–	–	–	–	–	–	–

**29**	**Procyanidin dimer B type isomer 3**	+	+	+	+	+	+	+	+	+	+	+	+
**30**	**Methyl 3-caffeoylquinate**	–	–	–	–	–	–	–	–	–	–	–	–
**31**	**Caffeic acid**	+	+	+	+	+	+	+	+	+	+	+	+
**32**	**Epicatechin**	+	+	+	+	+	+	–	–	–	–	–	–
**33**	**5-Caffeoylquinic acid isomer**	+	+	+	+	+	+	–	–	–	–	–	–
**34**	**Syringic acid**	+	+	+	+	+	+	–	–	–	–	–	–
**35**	**Caffeoylshikimic acid**	+	+	+	+	+	+	–	–	–	–	–	–
**36**	**Myricetin 3-*O*-rutinoside**	–	–	–	–	–	–	–	–	–	–	–	–
**37**	**Quercetin 3-*O*-hexoside-7-*O*-hexuronide**	–	–	–	–	–	–	–	–	–	–	–	–
**38**	**Myricetin 3-*O*-hexoside**	+	+	+	+	+	+	–	–	–	–	–	–
**39**	**Methyl 4-caffeoylquinate**	–	–	–	–	–	–	–	–	–	–	–	–
**40**	**Ellagic acid pentoside**	–	–	–	–	–	–	+	+	+	+	+	+
**41**	**Methyl 3-*p*-coumaroylquinate**	–	–	–	–	–	–	–	–	–	–	–	–
**42**	**Ellagic acid rhamnoside**	–	–	–	–	–	–	+	+	+	+	+	+
**43**	**Apigenin 8-*C*-glucoside**	+	+	+	+	+	+	–	–	–	–	–	–
**44**	**Methyl 5-caffeoylquinate isomer 1**	+	+	+	+	+	+	+	+	+	+	+	+
**45**	**Coumaric acid hexoside isomer 3**	–	–	–	–	–	–	+	+	+	+	+	+
**46**	**Quercetin 3-*O*-rutinoside**	+	+	+	+	+	+	–	–	–	–	–	–
**47**	**Myricetin 3-*O*-pentoside**	+	+	+	+	+	+	–	–	–	–	–	–
**48**	***p*-Coumaric acid**	+	+	+	+	+	+	+	+	+	+	+	+
**49**	**Quercetin 3-*O*-galactoside**	+	+	+	+	+	+	+	+	+	+	+	+
**50**	**Methyl 5-caffeoylquinate isomer 2**	+	+	+	+	+	+	–	–	–	–	–	–
**51**	**Ellagic acid**	–	–	–	–	–	–	+	+	+	+	+	+
**52**	**Kaempferol 7-*O*-rutinoside**	+	+	+	+	+	+	+	+	+	+	+	+
**53**	**Quercetin 3-*O*-rhamnosyl-hexuronide**	–	–	–	–	–	–	–	–	–	–	–	–
**54**	**Vanillic acid**	+	+	+	+	+	+	+	+	+	+	+	+
**55**	**Isorhamnetin 3-*O*-rutinoside**	+	+	+	+	+	+	–	–	–	–	–	–
**56**	**Quercetin 3-*O*-pentoside**	+	+	+	+	+	+	–	–	–	–	–	–
**57**	**Sinapic acid**	+	+	+	+	+	+	–	–	–	–	–	–
**58**	**Ferulic acid**	+	+	+	+	+	+	+	+	+	+	+	+
**59**	**Methyl 5-*p*-coumaroylquinate isomer 1**	–	–	–	–	–	–	–	–	–	–	–	–
**60**	**Kaempferol 3-*O*-glucoside**	+	+	+	+	+	+	+	+	+	+	+	+
**61**	**Syringetin 3-*O*-hexoside**	+	+	+	+	+	+	–	–	–	–	–	–

**62**	**Isorhamnetin 3-*O*-hexoside**	+	+	+	+	+	+	−	−	−	−	−	−
**63**	**Quercetin 3-*O*-acetyl-hexoside isomer 1**	+	+	+	+	+	+	−	−	−	−	−	−
**64**	**Isorhamnetin 3-*O*-hexuronide**	+	+	+	+	+	+	+	+	+	+	+	+
**65**	**Dicaffeoylquinic acid isomer 1**	+	+	−	+	+	−	−	−	−	−	−	−
**66**	**Methyl 5-*p*-coumaroylquinate isomer 2**	−	−	−	−	−	−	−	−	−	−	−	−
**67**	**Quercetin 3-*O*-acetyl-hexoside isomer 2**	+	+	+	+	+	+	−	−	−	−	−	−
**68**	**Quercetin 3-*O*-methyl-malonyl-hexoside**	+	+	+	+	+	+	−	−	−	−	−	−
**69**	**Quercetin 7-*O*-hexuronide**	+	+	+	+	+	+	+	+	+	+	+	+
**70**	**Isorhamnetin 3-*O*-pentoside**	+	+	+	+	+	+	−	−	−	−	−	−
**71**	**Quercetin 3-*O*-malonyl-hexoside**	+	+	+	+	+	+	+	+	+	+	+	+
**72**	**Dicaffeoylquinic acid isomer 2**	+	+	−	+	+	−	−	−	−	−	−	−
**73**	**Kaempferol 7-*O*-hexuronide**	−	−	−	−	−	−	+	+	+	+	+	+
**74**	**Isorhamnetin 3-*O*-malonyl-rutinoside**	+	−	−	+	−	−	−	−	−	−	−	−
**75**	**Myricetin**	−	−	−	+	+	+	−	−	−	−	−	−
**76**	**Methyl 3,4-dicaffeoylquinate**	+	−	−	+	−	−	−	−	−	−	−	−
**77**	**Kaempferol 3-*O*-hexuronide methyl ether**	+	+	+	+	+	+	+	+	+	+	+	+
**78**	**Kaempferol 3-*O*-malonyl-hexoside**	+	−	−	+	−	−	+	+	+	+	+	+
**79**	**Methyl caffeate**	+	+	+	+	+	+	+	+	+	+	+	+
**80**	**Methyl 3,5-dicaffeoylquinate**	+	+	−	+	+	−	−	−	−	−	−	−
**81**	**Feruloyl-coumaroylquinic acid isomer 1**	+	−	−	+	−	−	−	−	−	−	−	−
**82**	**Methyl 4,5-dicaffeoylquinate**	+	+	−	+	+	−	−	−	−	−	−	−
**83**	**Kaempferol 3-*O*-*p*-coumaroyl-hexoside**	−	−	−	−	−	−	+	+	+	+	+	+
**84**	***cis, trans*-Abscisic acid**	+	+	+	+	+	+	+	+	+	+	+	+
**85**	**Feruloyl-coumaroylquinic acid isomer 2**	+	+	−	+	+	−	−	−	−	−	−	−
**86**	**Quercetin**	+	+	+	+	+	+	+	+	+	+	+	+
**87**	**Feruloyl-coumaroylquinic acid isomer 3**	−	−	−	−	−	−	−	−	−	−	−	−
**88**	**Cinnamic acid**	+	+	+	+	+	+	+	+	+	+	+	+
**89**	**Naringenin**	+	+	+	+	+	+	−	−	−	−	−	−
**90**	**Kaempferol**	−	−	−	+	+	+	+	+	+	+	+	+
**91**	**Syringetin**	−	−	−	+	+	+	−	−	−	−	−	−
**92**	**Isorhamnetin**	−	−	−	+	+	+	−	−	−	−	−	−
**93**	**Pinocembrin**	−	−	−	−	−	−	−	−	−	−	−	−

+ stands for detected: − stands for not detected.

Table S2. Presence of each identified compound in integrated and organic strawberry and blueberry leaf samples.

No	Compound name	Blueberry-leaf						Strawberry-leaf					
		Integrated			Organic			Integrated			Organic		
		Bluecrop	Duke	Nui	Bluecrop	Duke	Nui	Alba	Favette	Clery	Alba	Favette	Clery
1	Gallic acid hexoside isomer 1	+	+	+	+	+	+	+	+	+	+	+	+
2	Dihydroxybenzoic acid hexoside isomer 1	+	+	+	+	+	+	+	+	+	+	+	+
3	Gallic acid hexoside isomer 2	+	+	+	+	+	+	+	+	+	+	+	+
4	Prodelphinidin dimer B type	+	+	+	+	+	+	–	–	–	–	–	–
5	Caffeoyltartaric acid	–	–	–	–	–	–	+	+	+	+	+	+
6	Chlorogenic acid hexoside isomer 1	+	+	+	+	+	+	+	+	+	+	+	+
7	Gallocatechin	+	+	+	+	+	+	–	–	–	–	–	–
8	Dihydroxybenzoic acid hexosyl-pentoside	+	+	+	+	+	+	+	+	+	+	+	+
9	Gallic acid hexoside isomer 3	+	+	+	+	+	+	+	+	+	+	+	+
10	Chlorogenic acid hexoside isomer 2	+	+	+	+	+	+	+	+	+	+	+	+
11	Caffeic acid hexoside isomer 1	+	+	+	+	+	+	–	–	–	–	–	–
12	Dihydroxybenzoic acid pentoside	+	+	+	+	+	+	+	+	+	+	+	+
13	3-*O*-Caffeoylquinic acid isomer 1	+	+	+	+	+	+	–	–	–	–	–	–
14	Hydroxybenzoic acid hexoside	–	–	–	–	–	–	+	+	+	+	+	+
15	3-*O*-Caffeoylquinic acid isomer 2	+	+	+	+	+	+	–	–	–	–	–	–
16	Procyanidin dimer B type isomer 1	+	+	+	+	+	+	+	+	+	+	+	+
17	Aesculin	+	+	+	+	+	+	+	+	+	+	+	+
18	Caffeic acid hexoside isomer 2	+	+	+	+	+	+	+	+	+	+	+	+
19	Coumaric acid hexoside isomer 1	+	+	+	+	+	+	+	+	+	+	+	+
20	Procyanidin dimer B type isomer 2	–	–	–	–	–	–	–	–	–	–	–	–
21	5-*O*-Caffeoylquinic acid	+	+	+	+	+	+	+	+	+	+	+	+
22	Epigallocatechin	–	–	–	–	–	–	–	–	–	–	–	–
23	Dihydroxybenzoic acid hexoside isomer 2	–	–	–	–	–	–	+	+	+	+	+	+
24	Quercetin 3-*O*-hexoside-7-*O*-hexuronide	–	–	–	–	–	–	+	+	+	+	+	+
25	Catechin	+	+	+	+	+	+	+	+	+	+	+	+
26	*p*-Hydroxybenzoic acid	+	+	+	+	+	+	+	+	+	+	+	+
27	Coumaric acid hexoside isomer 2	–	–	–	–	–	–	+	+	+	+	+	+
28	4-*O*-Caffeoylquinic acid	+	+	+	+	+	+	–	–	–	–	–	–

**29**	**Procyanidin dimer B type isomer 3**	–	–	–	–	–	–	–	–	–	–	–	–
**30**	**Methyl 3-caffeoylquinate**	+	+	+	+	+	+	–	–	–	–	–	–
**31**	**Caffeic acid**	+	+	+	+	+	+	+	+	+	+	+	+
**32**	**Epicatechin**	–	–	–	–	–	–	–	–	–	–	–	–
**33**	**5-Caffeoylquinic acid isomer**	+	+	+	+	+	+	+	+	+	+	+	+
**34**	**Syringic acid**	–	–	–	–	–	–	+	+	+	+	+	+
**35**	**Caffeoylshikimic acid**	+	+	+	+	+	+	+	+	+	+	+	+
**36**	**Myricetin 3-*O*-rutinoside**	+	+	+	+	+	+	–	–	–	–	–	–
**37**	**Quercetin 3-*O*-hexosyl-hexuronide**	–	–	–	–	–	–	+	+	+	+	+	+
**38**	**Myricetin 3-*O*-hexoside**	+	+	+	+	+	+	–	–	–	–	–	–
**39**	**Methyl 4-caffeoylquinate**	+	+	+	+	+	+	–	–	–	–	–	–
**40**	**Ellagic acid pentoside**	–	–	–	–	–	–	+	+	+	+	+	+
**41**	**Methyl 3-*p*-coumaroylquinate**	+	+	+	+	+	+	–	–	–	–	–	–
**42**	**Ellagic acid rhamnoside**	–	–	–	–	–	–	+	+	+	+	+	+
**43**	**Apigenin 8-*C*-glucoside**	+	+	+	+	+	+	–	–	–	–	–	–
**44**	**Methyl 5-caffeoylquinate isomer 1**	+	+	+	+	+	+	+	+	+	+	+	+
**45**	**Coumaric acid hexoside isomer 3**	–	–	–	–	–	–	+	+	+	+	+	+
**46**	**Quercetin 3-*O*-rutinoside**	+	+	+	+	+	+	–	–	–	–	–	–
**47**	**Myricetin 3-*O*-pentoside**	+	+	+	+	+	+	–	–	–	–	–	–
**48**	***p*-Coumaric acid**	+	+	+	+	+	+	+	+	+	+	+	+
**49**	**Quercetin 3-*O*-galactoside**	+	+	+	+	+	+	+	+	+	+	+	+
**50**	**Methyl 5-caffeoylquinate isomer 2**	+	+	+	+	+	+	–	–	–	–	–	–
**51**	**Ellagic acid**	–	–	–	–	–	–	+	+	+	+	+	+
**52**	**Kaempferol 7-*O*-rutinoside**	+	+	+	+	+	+	–	–	–	–	–	–
**53**	**Quercetin 3-*O*-rhamnosyl-hexuronide**	–	–	–	–	–	–	+	+	+	+	+	+
**54**	**Vanillic acid**	+	+	+	+	+	+	+	+	+	+	+	+
**55**	**Isorhamnetin 3-*O*-rutinoside**	+	+	+	+	+	+	–	–	–	–	–	–
**56**	**Quercetin 3-*O*-pentoside**	+	+	+	+	+	+	+	+	+	+	+	+
**57**	**Sinapic acid**	+	+	+	+	+	+	+	+	+	+	+	+
**58**	**Ferulic acid**	+	+	+	+	+	+	+	+	+	+	+	+
**59**	**Methyl 5-*p*-coumaroylquinate isomer 1**	+	+	+	+	+	+	–	–	–	–	–	–
**60**	**Kaempferol 3-*O*-glucoside**	+	+	+	+	+	+	+	+	+	+	+	+
**61**	**Syringetin 3-*O*-hexoside**	+	+	–	+	+	–	–	–	–	–	–	–

**62**	**Isorhamnetin 3-*O*-hexoside**	+	+	+	+	+	+	−	−	−	−	−	−
**63**	**Quercetin 3-*O*-acetyl-hexoside isomer 1**	+	+	+	+	+	+	−	−	−	−	−	−
**64**	**Isorhamnetin 3-*O*-hexuronide**	+	+	+	+	+	+	+	+	+	+	+	+
**65**	**Dicaffeoylquinic acid isomer 1**	+	+	−	+	+	−	−	−	−	−	−	−
**66**	**Methyl 5-*p*-coumaroylquinate isomer 2**	+	+	+	+	+	+	−	−	−	−	−	−
**67**	**Quercetin 3-*O*-acetyl-hexoside isomer 2**	+	+	+	+	+	+	−	−	−	−	−	−
**68**	**Quercetin 3-O-methyl-malonyl-hexoside**	+	+	+	+	+	+	−	−	−	−	−	−
**69**	**Quercetin 7-*O*-hexuronide**	+	+	+	+	+	+	+	+	+	+	+	+
**70**	**Isorhamnetin 3-*O*-pentoside**	−	+	−	−	+	−	−	−	−	−	−	−
**71**	**Quercetin 3-*O*-malonyl-hexoside**	+	+	+	+	+	+	−	−	−	−	−	−
**72**	**Dicaffeoylquinic acid isomer 2**	+	+	−	+	+	−	−	−	−	−	−	−
**73**	**Kaempferol 7-*O*-hexuronide**	−	−	−	−	−	−	+	+	+	+	+	+
**74**	**Isorhamnetin 3-*O*-malonyl-rutinoside**	+	+	−	+	+	−	−	−	−	−	−	−
**75**	**Myricetin**	+	+	+	+	+	+	−	−	−	−	−	−
**76**	**Methyl 3,4-dicaffeoylquinate**	+	−	−	+	−	−	−	−	−	−	−	−
**77**	**Kaempferol 3-*O*-hexuronide methyl ether**	−	−	−	−	−	−	+	+	+	+	+	+
**78**	**Kaempferol 3-*O*-malonyl-hexoside**	+	+	+	+	+	+	−	−	−	−	−	−
**79**	**Methyl caffeate**	+	+	+	+	+	+	+	+	+	+	+	+
**80**	**Methyl 3,5-dicaffeoylquinate**	+	+	−	+	+	−	−	−	−	−	−	−
**81**	**Feruloyl-coumaroylquinic acid isomer 1**	+	−	−	+	−	−	−	−	−	−	−	−
**82**	**Methyl 4,5-dicaffeoylquinate**	+	+	−	+	+	−	−	−	−	−	−	−
**83**	**Kaempferol 3-*O*-*p*-coumaroyl-hexoside**	−	−	−	−	−	−	+	+	+	+	+	+
**84**	***cis, trans*-Abscisic acid**	−	−	−	−	−	−	−	−	−	−	−	−
**85**	**Feruloyl-coumaroylquinic acid isomer 2**	+	+	−	+	+	−	−	−	−	−	−	−
**86**	**Quercetin**	+	+	+	+	+	+	+	+	+	+	+	+
**87**	**Feruloyl-coumaroylquinic acid isomer 3**	+	+	−	+	+	−	−	−	−	−	−	−
**88**	**Cinnamic acid**	+	+	+	+	+	+	+	+	+	+	+	+
**89**	**Naringenin**	+	+	+	+	+	+	+	+	+	+	+	+
**90**	**Kaempferol**	+	+	+	+	+	+	+	+	+	+	+	+
**91**	**Syringetin**	−	−	−	−	−	−	−	−	−	−	−	−
**92**	**Isorhamnetin**	+	+	+	+	+	+	−	−	−	−	−	−
**93**	**Pinocembrin**	−	−	−	−	−	−	+	+	+	+	+	+

+ stands for detected: − stands for not detected.

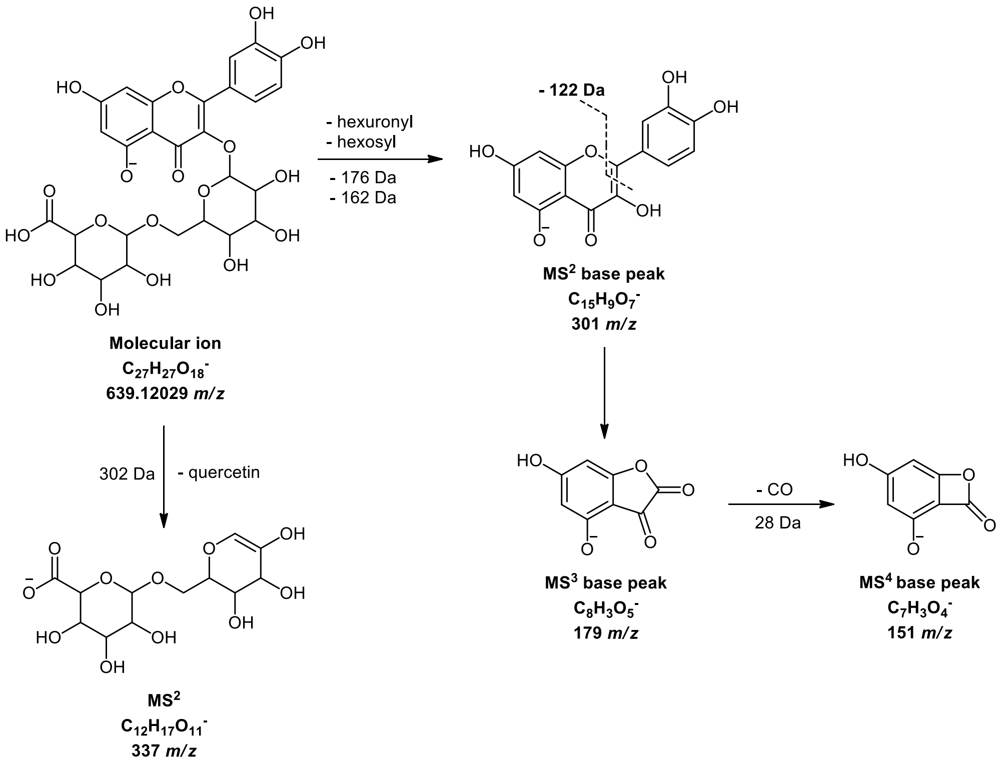

Figure S1. Proposed fragmentation pathway of compound **37**.
